# An Insight Into the microRNA Profile of the Ectoparasitic Mite *Varroa destructor* (Acari: Varroidae), the Primary Vector of Honey Bee Deformed Wing Virus

**DOI:** 10.3389/fcimb.2022.847000

**Published:** 2022-03-16

**Authors:** Deepak Kumar, Mohamed Alburaki, Faizan Tahir, Michael Goblirsch, John Adamczyk, Shahid Karim

**Affiliations:** ^1^ School of Biological, Environmental, and Earth Sciences, University of Southern Mississippi, Hattiesburg, MS, United States; ^2^ Bee Research Laboratory, Beltsville, United States Department of Agriculture, Agricultural Research Service (USDA ARS), Beltsville, MD, United States; ^3^ Southern Horticultural Research Unit, USDA ARS, Poplarville, MS, United States; ^4^ Center for Molecular and Cellular Biology, University of Southern Mississippi, Hattiesburg, Hattiesburg, MS, United States

**Keywords:** *Varroa destructor*, microRNAs, small RNA-seq, honey bee (*Apis mellifera L.*), deformed wing virus

## Abstract

The remarkably adaptive mite *Varroa destructor* is the most important honey bee ectoparasite. *Varroa* mites are competent vectors of deformed wing virus (DWV), and the *Varroa*-virus complex is a major determinant of annual honey bee colony mortality and collapse. MicroRNAs (miRNAs) are 22-24 nucleotide non-coding RNAs produced by all plants and animals and some viruses that influence biological processes through post-transcriptional regulation of gene expression. Knowledge of miRNAs and their function in mite biology remains limited. Here we constructed small RNA libraries from male and female *V. destructor* using Illumina’s small RNA-Seq platform. A total of 101,913,208 and 91,904,732 small RNA reads (>18 nucleotides) from male and female mites were analyzed using the miRDeep2 algorithm. A conservative approach predicted 306 miRNAs, 18 of which were upregulated and 13 downregulated in female *V. destructor* compared with males. Quantitative real-time PCR validated the expression of selected differentially-expressed female *Varroa* miRNAs. This dataset provides a list of potential miRNA targets involved in regulating vital *Varroa* biological processes and paves the way for developing strategies to target *Varroa* and their viruses.

## Introduction

*Varroa destructor* mites are considered the most damaging ectoparasite of the European honey bee (*Apis mellifera*) (Hristov et al., 2020), with consequent colony losses threatening global honey production (Gallai et al., 2009; Le Conte et al., 2010; Goulson et al., 2015; Hung et al., 2018; Noël et al., 2020). *Varroa* mites infest honey bees, where they feed on the hemolymph and fat body of pupae and adults (Ramsey et al., 2019), depleting vital nutritional resources and suppressing the immune system in host bees (Yang and Cox-Foster, 2005; Di Prisco et al., 2016; Koleoglu et al., 2017; Koleoglu et al., 2018; Annoscia et al., 2019). *Varroa* are competent vectors of various viral pathogens (Gisder et al., 2009; de Miranda and Genersch, 2010; Wilfert et al., 2016), which together contribute to increased winter losses through colony collapse in many regions (Highfield et al., 2009) and requiring significant investment from beekeepers to combat infestations and replace stock. Typically, *Varroa*-infested hives will not survive for more than three years without treatment or cultural intervention (Martin, 1998; van Dooremalen et al., 2012). Indeed, in the United States, chemical treatment of honey bee colonies improves colony survival (Webster and Delaplane, 2001; Kulhanek et al., 2021). Although accurately costing the losses to honey bee colonies caused by *Varroa* infestation is technically challenging, it is safe to assume that Varroa infestation has resulted in the collapse of many thousands of honeybee colonies and billions of dollars of losses (Rosenkranz et al., 2010).

*Varroa* foundresses vector deformed wing virus (DWV), one of the major causes of honey bee colony collapse, to immature honey bees when they feed. There is now considerable evidence that *Varroa* mites are a vector of DWV (Santillán-Galicia et al., 2010; Gisder et al., 2018; Posada-Florez et al., 2019; Ryabov et al., 2019), further contributing to the negative impact of the mite on honey bees (Rosenkranz et al., 2010; Yañez et al., 2020). While DWV infection is usually commensal in honey bees and rarely pathogenic in the absence of *Varroa* infestation (Beaurepaire et al., 2019), inoculation of DWV during *Varroa* feeding significantly increases viral load in the pupal stage (Gusachenko et al., 2020). Bees symptomatic for DWV emerge with wing deformities, have reduced weight, and have a shortened lifespan or are killed prematurely by other colony members. Although bees can be asymptomatic, high viral loads adversely impact lifespan, foraging and flight capability, behavioral maturation, and immunity (Iqbal and Mueller, 2007; Natsopoulou et al., 2016; Wells et al., 2016; Benaets et al., 2017; Brettell et al., 2017; Traniello et al., 2020; Pizzorno et al., 2021). By feeding on honey bee pupae, *Varroa* act as an efficient vector of over a dozen viruses to their bee hosts. DWV load in the hive significantly increases upon *Varroa* infestation, subsequently impacting honey bee mortality (Beaurepaire et al., 2020; Gusachenko et al., 2020). These *Varroa*-borne viruses have several co-circulating variants that differ in virulence (Moore et al., 2011; De Miranda et al., 2013; Mordecai et al., 2016; Gisder et al., 2018; Martin and Brettell, 2019; Posada-Florez et al., 2019; Remnant et al., 2019; Ryabov et al., 2019). Although there is a clear association between DWV in *Varroa* and subsequent infection of honey bees with the virus, the underlying genetic mechanisms of vector competence (acquisition, maintenance, and transmission) need further clarification to develop interventions to prevent *Varroa* infestation and *Varroa*-borne virus transmission to honey bees.

The highly efficient vectoring of honey bee viruses by *Varroa* contributes to driving changes in virus distribution, prevalence, and virulence (Traynor et al., 2020). *Varroa* control and prevention are primarily based on chemical acaricides, but these contribute to *Varroa* resistance (Higes et al., 2020; Rinkevich, 2020), and novel, effective, and safe approaches are clearly needed to tackle this global problem. To this end, here we investigated microRNAs (miRNAs) that might regulate *Varroa* vector competence and therefore act as targets to disrupt pathogen transmission. MicroRNAs are single stranded non-coding RNAs 22-25 nucleotides in length derived from larger hairpin RNA precursors. MicroRNAs play significant roles in pathophysiological post-transcriptional regulation of their target genes.

MicroRNAs are now established therapeutic targets in several diseases (Bartel, 2018). In mammals, miRNAs regulate 30-60% of protein-coding genes and most cellular processes (Filipowicz et al., 2008; Yu and Pan, 2012). However, information on arthropod miRNAs and their role in arthropod physiology is limited (Barrero et al., 2011; Lucas and Raikhel, 2013; Luhur et al., 2013; Zhou et al., 2013; Asgari, 2014; Blair and Olson, 2015; He et al., 2015; Luo et al., 2015; Shao et al., 2015; Wang et al., 2015). MicroRNAs are known to play a significant role in the establishment of sexual dimorphism and in controlling sexual behavior (Lucas et al., 2015a), and the presence of male- and female-biased miRNAs and ovary- and testis-enriched miRNAs suggest a role in sex determination and gametogenesis (Fagegaltier et al., 2014). For example, dme-let-7 depletion delays germline differentiation of the early ovary, disrupts aggregation of somatic cells in the testes, and plays a significant role in sexual identity (male or female) during the late-larval to late-pupal stages and during throughout adulthood *via* ecdysone signaling (Fagegaltier et al., 2014). In addition, dme-miR-124 controls male sexual differentiation by targeting sex-specific splicing factor in the sex determination pathway (Weng et al., 2013). Male flies with mutant dme-miR-124 have aberrant pheromone levels, reducing mating success and receptivity by females and increasing male-male courtship. Furthermore, miRNA expression altered in different tissues post-mating following receipt of sex peptide (SP) from male flies (Fricke et al., 2014). Female flies lacking specific miRNAs (such as dme-miR-184, dme-miR-279, dme-miR-278, and dme-miR-317) demonstrated abnormal receptivity to SP from male flies (Lucas et al., 2015a).

Given the importance of *Varroa* as a honey bee parasite and vector of honey bee viruses, the recent availability of high-quality *de novo* reference genomes for *V. destructor* and *V. jacobsoni* has opened up new avenues to map non-coding small RNAs in the mite genome (Techer et al., 2019). MicroRNAs are now known to not only be critical regulators of many biological processes and pathogen infections in arthropods (Behura, 2007; Feng et al., 2018), but also play a role in replicating, harboring, and inhibiting other RNA viruses such as dengue virus (DENV), chikungunya virus (CHIKV), and other viruses and intracellular pathogens in arthropods (Sempere et al., 2003; Hussain et al., 2013; Maharaj et al., 2015). Therefore, *Varroa* microRNAs may also play a significant role in DWV colonization and its transmission to honey bees.

The aim of this study was to predict the repertoire of conserved and novel miRNAs in male and female *Varroa* to provides insights into the pathophysiological roles of *Varroa* miRNAs, their gene regulatory networks, and their potential biological functions. The identification of novel miRNAs in *Varroa* paves the way for an improved understanding of the role played by miRNAs in the transmission of *Varroa*-borne viruses and potential targets for the control and prevention of mites and viruses in bee colonies.

## Materials and Methods

### *Varroa* Mite Collection

*Varroa* mites were collected from the honey bee apiary at the University of Southern Mississippi’s Lake Thoreau Environmental Center, Hattiesburg, MS (31° 20’ 54.476” N and 89° 25’ 9.202” W) between August and October, 2019. The apiary comprises five healthy and well-established Italian *Apis mellifera ligustica* colonies that had successfully survived the mild winter in MS the previous year (2018) and that had not received *Varroa* treatment of any kind in 2019 prior to the sampling period. Capped brood and mites were collected from a single-well populated queen-right colony with two chambers hosted in a classic Langstroth hive. Adult female mites were collected from adult honey bee workers using the sugar shake method (Gregorc et al., 2018). Male *Varroa were* collected from sealed pupal cells by removing the wax cap and positively identifying males in infested cells by microscopy. Ten male and 20 female *Varroa* were collected separately in tubes containing Trizol for RNA extraction. Infection with DWV serotypes A, B, or C was determined using specific primers as described in Kevill et al. (2017).

### RNA Extraction

RNA was extracted from male and female mites separately using the Trizol extraction method (Chomczynski and Mackey, 1995) with some modifications to the original protocol. Briefly, mites were collected individually and then male and female mites were pooled in separate tubes. Mites were homogenized in 500 µl Trizol using a plastic pestle. After homogenization, samples were mixed for 10 min at room temperature in a shaker followed by centrifugation at 15000 x g for 10 min at 4°C. The supernatant was transferred to a new tube and incubated for 5 min at room temperature to permit complete dissociation of the nucleoproteins. One hundred µl chilled chloroform was added to the samples, mixed, and incubated for 10 min at 4°C. Samples were centrifuged at 15000 x g for 15 mins to obtain an aqueous phase. The aqueous phase was transferred to a new tube and then 600 µl of isopropanol was added before storage overnight at -20°C. The following day, samples were centrifuged for 15 min at 4°C followed by washing the pellets in 70% ethanol. RNA pellets were dried, and RNA samples were resuspended in sterile water and quantified using a NanoDrop instrument (Thermo Fisher Scientific, Waltham, MA).

### Small RNA Sequencing

The RNA concentration was 286.5 ng/ul (260/280 = 1.8) for pooled *Varroa* females and 185.6 ng/ul (260/280 = 1.8) for pooled *Varroa* males. Small RNA libraries were prepared using the Illumina TruSeq kit following the manufacturer’s instructions (Illumina, San Diego, CA). Briefly, short adapter oligonucleotides were ligated to each end of the small RNAs in the samples. Individual cDNA copies were made with reverse transcriptase, and PCR was used to add sample-specific barcodes and Illumina sequencing adapters. The final concentration of all NGS libraries was determined using a Qubit fluorometric assay. The cDNA fragment size of each library was assessed using a DNA 1000 high-sensitivity chip on an Agilent 2100 Bioanalyzer (Agilent Technologies, Santa Clara, CA). Before sequencing, samples passed the essential quality control (QC) test. After purification by polyacrylamide gel electrophoresis, the sample libraries were pooled and sequenced on an Illumina NextSeq 500 (300 cycles, single-end, 36 bases) using the TruSeq SBS kit v3 (Illumina) and protocols defined by the manufacturer. Four small RNA libraries of pooled male and female *Varroa* mites were sequenced *via* Illumina small RNA high-throughput sequencing. RNA library preparation and indexing were performed by the University of Mississippi Medical Center genomics core facility.

### Bioinformatics Analysis

The miRDeep2 version 2.0.0.8 software package (Friedländer et al., 2008; Friedländer et al., 2012) was used to process the sequencing data. The reads from all samples were combined for novel miRNA prediction. The mapper function of miRDeep2 was used to trim the adapter sequences from the reads and convert the read files from FASTQ to FASTA format. Reads shorter than 18 bases were discarded. Remaining reads were then mapped to the *V. destructor* reference genome (GCF_002443255.1_Vdes_3.0_genomic.fna) (Techer et al., 2019) using the default miRDeep2 mapper function parameters. Reads mapping to the genome were used to predict novel miRNAs. The *Drosophila melanogaster* genome was also provided as a reference genome, and mapped reads were aligned to available miRNAs of *D. melanogaster* in miRbase v22 and quantified. The output file included the sequence and location of the possible miRNAs, the number of reads mapping to them, and a score reflecting the likelihood that the predicted miRNA was not due to chance alone. The software aligned the reads to the reference genomes of *D. melanogaster* and *V. destructor* and looked for locations where potential miRNA reads accumulated. The regions immediately surrounding the mapped reads were examined for miRNA biogenesis features including mature miRNAs, star and precursor reads, and stem-loop folding properties. The miRDeep2 program models the miRNA biogenesis pathway using a probabilistic algorithm to score compatibility of the position and frequency of next-generation sequencing (NGS) reads with the secondary structure of the miRNA precursor. BEDtools (Quinlan and Hall, 2010) was used to determine the genomic origin of the precursors with the variant intersect.

### Validation of Differentially-Expressed miRNAs by qRT-PCR

The miRprimer2 algorithm (Busk, 2014) was used to design qRT-PCR primers for the predicted miRNAs. Predicted miRNAs differentially expressed in small RNA sequencing data were validated by qRT-PCR. RNA samples from seven male and eleven female *Varroa* were separately pooled to extract RNA for cDNA synthesis, and the miRNA-specific qRT-PCR reaction was performed for each sample. The Mir-X miRNA qRT-PCR TB Green kit from Takara Bio (Kusatsu, Shiga, Japan; catalog # 638316) was used for cDNA synthesis and miRNA expression analysis. Conditions for qRT-PCR were: initial denaturation 95°C for 10 mins and then 40 cycles of 95°C for 5 secs and 60°C for 20 secs on a C1000 Touch Thermal cycler (Bio-Rad Laboratories, Hercules, CA; CFX96 Real-Time System). The primer sequences are shown in **Supplementary Table S1**.

### Normalization, Differential Expression, and Statistical Analysis of miRNA Expression in Male and Female Varroa Mites

*In silico* differential expression (DE) analysis of predicted miRNAs was performed using the DeApp interactive web interface (Li and Andrade, 2017). DeApp is a web-based, graphical interface developed in R with the *shiny* package (web application framework for R). Low expression genetic features were removed after alignment if the counts per million (CPM) value was ≤1 in less than two samples. Sample normalization and a multidimensional scaling (MDS) plot are shown in **Supplementary Figure S1** and include details about sample distribution after filtering out low expression genomic features. Differential expression (DE) analysis was performed using edgeR with the false discovery rate (FDR)-adjusted *p*-value set to 0.05 and minimum fold-change of 1.5. The interface displays a dispersion plot showing the results of overall DE analysis along with statistical significance (*p*-value, FDR adjusted *p*-value) and volcano plot corresponding to the specified parameters and cutoff values.

### Prediction of the Target Genes, Proteome Re-Annotation, and Gene Ontology (GO) and KEGG Enrichment Analyses

The targeting algorithms TargetSpy (Sturm et al., 2010), MIRANDA (John et al., 2004), and PITA (Kertesz et al., 2007) were used in miRNAcons Target from sRNAtoolbox to predict the genes regulated by up- or downregulated *Varroa* miRNAs (Aparicio-Puerta et al., 2019). Targets common in all three programs were further considered. *In silico* target prediction resulted in a high number of false positives, but cross-species comparisons and combinatorial effects reduced this number (Min and Yoon, 2010). Lists of target genes were functionally characterized using the STRING webserver (Franceschini et al., 2013). The networks of target genes and the KEGG pathways significantly enriched for target genes were extracted using the STRING output (**Table 3**). PANNZER2 (Toronen et al., 2018) was used to functionally re-annotate the predicted proteome of up- or downregulated genes (targets of predicted miRNAs), and WEGO (Ye et al., 2006; Ye et al., 2018) was used to analyze and plot gene ontology (GO) annotations.

## Results

### Read Length Distribution of Small RNAs

**Figure 1:**
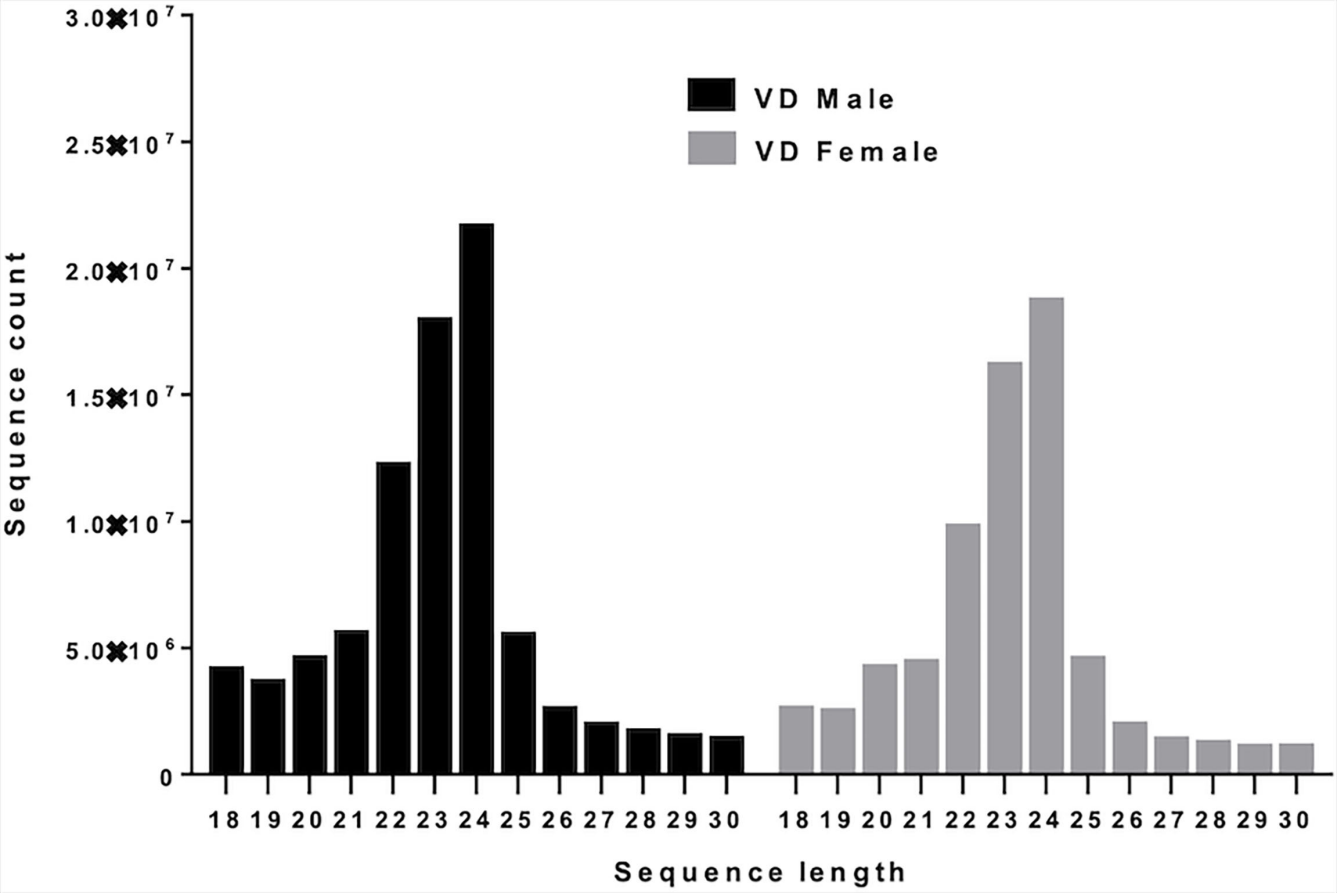
Small RNA sequence length distribution of microRNAs in male and female *Varroa destructor* (VD) mites. MicroRNAs are twenty-four (24) nucleotides in length.

After adapter trimming and removal of short reads (≤18 nucleotides), 101,914,732 small RNA reads were available from *Varroa* males and 91,904,732 from females for downstream analysis. 27,204,163 male and 32,283,353 female *Varroa* reads matched to the *Varroa* genome. The read length distribution indicates the types of small RNAs present in male and female *Varroa* samples; **Figure 1** depicts the number of short read sequences between 18 and 30 nucleotides, and three main peaks were distinguishable in both male and female samples: (i) a peak at 24 nucleotides, the highest abundance population of mature miRNAs; and (ii) relatively less abundant peaks at 22 and (iii) 23 nucleotides. There were ~2.2 x 10^7^ and ~1.8 x 10^7^ 24 nucleotide miRNA sequences in male and female *Varroa*, respectively, and ~1.25 x 10^7^ and ~1.8 x 10^7^ 22 and 23 nucleotide small RNA sequences in males and ~1.0 x10^7^ and ~1.7 x 10^7^ 22 and 23 nucleotide small RNA sequences in females, respectively. The read length distribution in male *Varroa* was qualitatively identical to females.

### MicroRNA Profiles of *Varroa*

**Figure 2:**
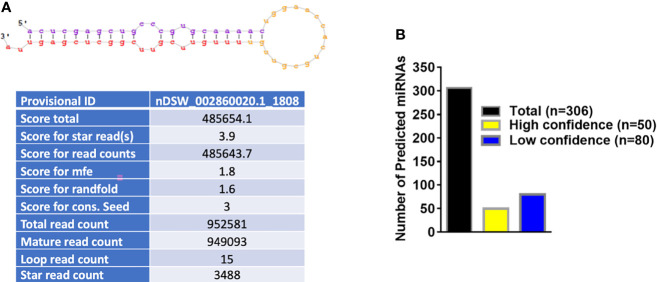
**(A)** Basic stem-loop structure of a predicted microRNA. miRDeep2 was used to identify potential miRNA precursors based on nucleotide length, star sequence, stem-loop folding, and homology to the *Varroa* reference genome. Shown are the predicted stem-loop structures (yellow), star (violet), and mature sequences (red). **(B)** Annotation of predicted *Varroa destructor* microRNAs. 306 microRNAs were predicted in *Varroa* samples. 50 were categorized as high-confidence and 80 as low-confidence miRNAs based on standard criteria.

**Figure 3:**
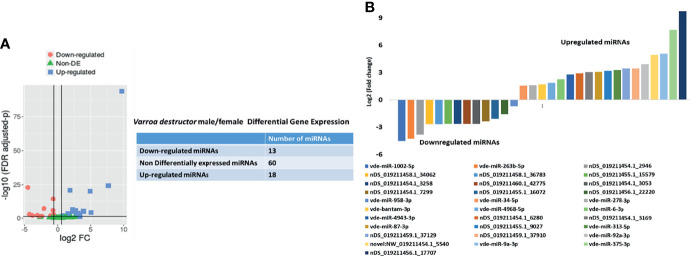
**(A)** *In silico* differential expression analysis of predicted microRNAs in female *Varroa destructor* relative to males. EdgeR was used for differential expression analysis. 13 predicted microRNAs were downregulated, 18 upregulated, and 60 were unaffected. **(B)** Differential expression of individual microRNAs in *Varroa* females relative to males. miRNAs with a log2 fold-change expression > |1| and FDR ≤ 0.1 were considered significantly differentially expressed with respect to male miRNAs (refer to **Table 1**).

**Figure 2A** shows the basic hairpin loop structure of a microRNA and other parameters (dicer cut overhangs, total read count, mature read count, loop read count, total read count, randfold score, and total score) used to determine whether a hairpin loop-structured RNA is an miRNA. A total of 306 microRNAs were predicted in *V. destructor*, and several had homologs in *D. melanogaster.* The predicted miRNAs were categorized as high (n=50) and low confidence (n=80) miRNAs (**Figure 2B** and **Table S2**) based on standard criteria (Bartel, 2018). Eighteen of the predicted miRNAs were upregulated and 13 were downregulated in female *Varroa* compared with male *Varroa* (**Figures 3A, B** and **Table 1**). Two of the predicted mature microRNAs, nDS_019211455.1_10989 and nDS_019211455.1_16072, were male-specific, and three of the predicted miRNAs (nDS_019211455.1_10999, nDS_019211459.1_37116, and nDS_019211455.1_10993; **Table S1**, **S3**) were female-specific. **Supplementary Table S2** includes the consensus mature, star, total read counts, and miRDeep2 scores and probabilities for every miRDeep2-predicted miRNA with an miRDeep2 score.

**Table 1:** *In silico* differential expression analysis of whole-body female *Varroa* mite microRNAs relative to male microRNAs.

Predicted miRNA	log2FC	logCPM	LR	P-value	FDR
vde-miR-1002-5p	-4.511	13.92626	108.6748	1.9E-25	5.8E-24
vde-miR-263b-5p	-4.27	11.69984	13.43531	0.00025	0.00125
novel:nDS_019211454.1_2946	-3.814	11.56138	9.607184	0.00194	0.0084
novel:nDS_019211458.1_34062	-2.671	11.8175	10.05673	0.00937	0.03157
novel:nDS_019211458.1_36783	-2.671	11.69432	6.75152	0.00937	0.03157
novel:nDS_019211455.1_15579	-2.639	11.69432	6.75152	0.04995	0.11365
novel:nDS_019211454.1_3258	-2.639	11.87389	7.292297	0.04995	0.11365
novel:nDS_019211460.1_42775	-2.639	13.6527	33.64388	0.04995	0.11365
novel:nDS_019211454.1_3053	-2.639	13.59985	9.317998	0.04995	0.11365
novel:nDS_019211454.1_7299	-2.37	13.59696	6.516879	0.00693	0.02524
novel:nDS_019211455.1_16072	-2.079	16.93765	27.64951	6.6E-09	8.6E-08
novel:nDS_019211456.1_22220	-1.586	17.49914	65.70939	0.00049	0.00236
vde-miR-958-3p	-0.714	15.89762	12.14634	1.5E-07	1.5E-06
vde-miR-34-5p	1.544	14.39795	8.821865	5.7E-05	0.00033
vde-miR-278-3p	1.616	13.43659	5.955626	0.00693	0.02524
vde-bantam-3p	1.678	13.71432	16.20957	5.2E-16	7.9E-15
vde-miR-4968-5p	1.844	12.6881	7.289986	4.5E-23	1E-21
vde-miR-6-3p	2.249	15.67949	97.85967	1.3E-06	9.5E-06
vde-miR-4943-3p	2.781	13.41013	23.49429	4E-07	3.7E-06
novel:nDS_019211454.1_6280	2.903	13.71203	30.86426	0.04952	0.11365
novel:nDS_019211454.1_3169	3.035	13.19444	25.68681	3.6E-05	0.00024
vde-miR-87-3p	3.064	12.65187	16.14825	0.00152	0.00691
novel:nDS_019211455.1_9027	3.186	12.73314	17.05458	0.0281	0.07748
vde-miR-313-5p	3.276	12.38077	14.03693	0.00018	0.00096
novel:nDS_019211459.1_37129	3.422	11.60818	5.786587	0.01615	0.0474
novel:nDS_019211459.1_37910	3.422	11.60899	5.786596	0.01615	0.0474
vde-miR-92a-3p	3.915	12.76538	23.93004	1E-06	8.3E-06
novel:nDS_019211454.1_5540	4.923	14.12569	93.45913	4.1E-22	7.5E-21
vde-miR-9a-3p	5.062	12.30162	19.28666	1.1E-05	7.9E-05
vde-miR-375-3p	7.668	14.18509	114.5216	1E-26	4.6E-25
novel:nDS_019211456.1_17707	9.727	16.02422	433.5094	2.8E-96	2.5E-94

miRNAs with a log2 fold-change in expression > |1| and FDR ≤ 0.1 were considered significantly differentially expressed with respect to male microRNAs.

### *In Silico* Mapping of *Varroa* Small RNA Sequences to *Apis mellifera* and DWV-B (Deformed Wing Virus) Genomes

*In silico* mapping of *Varroa* small RNA sequences to the *Apis mellifera* genome (GCF_003254395.2_Amel_HAv3.1_genomic.fna) detected 112 miRNA orthologs (**Table S4**). We used the DWV-B genome (GCF_000856945.1_ViralProj15121_genomic.fna.gz; https://www.ncbi.nlm.nih.gov/assembly/GCF_000856945.1/) as the DWV reference. *In silico* mapping of female *Varroa* small RNA sequences suggested that female samples were DWV-infected, since 0.18% of female *Varroa* mite sequences mapped to the DWV-B genome. The few viral miRNAs predicted in the analysis had very low mIRDeep2 scores so could not be regarded as predicted viral miRNAs.

### MicroRNA Differential Expression Analysis by qRT-PCR

**Figure 4:**
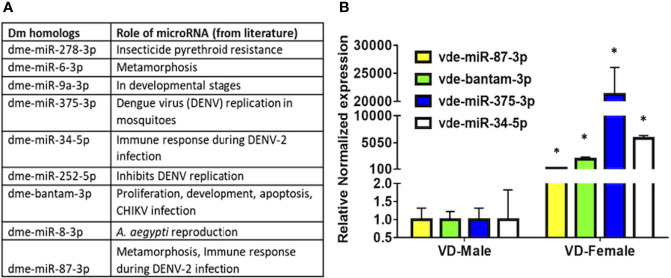
**(A)** MicroRNA candidates predicted in this study, conserved in other arthropods, and validated in the available literature play significant roles in replication, harboring, and inhibition of viral pathogens. **(B)** qRT-PCR expression of potential microRNAs found in the present study in DWV-B-infected male and female *Varroa* mites. Succinate dehydrogenase (*SDHA*) was used as the housekeeping gene for normalization. Expression of these microRNAs is comparatively higher in female than male *Varroa*. Statistical significance for qRT-PCR-based differential expression was determined using the two-tailed Student’s *t*-test, where * denotes *p* < 0.05.

Several of the predicted *Varroa* miRNAs were conserved in *D. melanogaster*, and previous studies revealed their conserved roles in development, replication, viral colonization, and viral inhibition in other arthropods (**Figure 4A** and **Table 2**). These high-confidence predicted miRNAs such as vde-miR-87-3p, vde-bantam-3p, vde-miR-375-3p, and vde-miR-34-5p were upregulated 100- to 20,000-fold in DWV-infected female relative to male *Varroa* by qRT-PCR analysis (**Figure 4B**).

**Table 2:** List of potential microRNA candidates predicted in this study, conserved in *Drosophila melanogaster* (Dm), and with evidence in the available literature play a significant role in the development, replication, harboring, and inhibition of viral pathogens.

Predicted microRNA	Annotation	Dm homologs	Role of microRNA (from literature)	Target genes	Reference
vde-miR-278-3p	High Confidence	dme-miR-278-3p	Insecticide pyrethroid resistance	*CYP6AG11*	Lei et al., 2015
vde-miR-6-3p	High Confidence	dme-miR-6-3p	Metamorphosis		Sempere et al., 2003
vde-miR-9a-3p	High Confidence	dme-miR-9a-3p	In developmental stages		
vde-miR-375-3p	High Confidence	dme-miR-375-3p	Dengue virus (DENV) replication in mosquitoes	*Cactus, REL1*	Hussain et al., 2013
vde-miR-34-5p	High Confidence	dme-miR-34-5p	Immune response during DENV-2 infection		Liu et al., 2015
vde-miR-252-5p	High Confidence	dme-miR-252-5p	Inhibits DENV replication		Yan et al., 2014
vde-bantam-3p	High Confidence	dme-bantam-3p	Proliferation, development, apoptosis, CHIKV infection		Maharaj et al., 2015
vde-miR-8-3p	High Confidence	dme-miR-8-3p	A. aegypti reproduction	*SWIM*	Lucas et al., 2015a
vde-miR-87-3p	High Confidence	dme-miR-87-3p	Metamorphosis, Immune response during DENV-2 infection		Sempere et al., 2003

### Other Small RNA Categories

**Figure 5:**
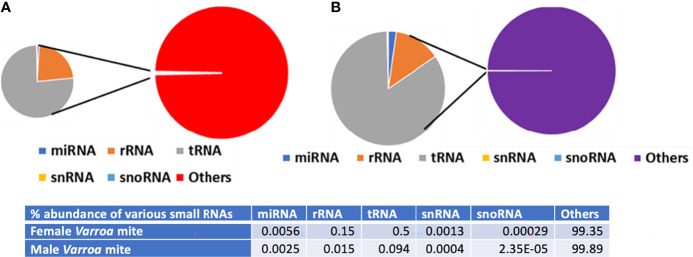
Summary of *Varroa*-derived female **(A)** and male **(B)** small RNA reads matching various small RNA categories.

A summary of reads from both male and female *Varroa* matching various small RNA categories is shown in **Figure 5**. Other small RNAs include signal recognition particle (SRP), protein-coding, and not annotated RNAs. Of the total small RNA reads from female *Varroa*, 56 x 10^-4^% were miRNAs, 15 x 10^-2^ were rRNAs, 5 x 10^-1^ were tRNAs,13 x 10^-4^ were snRNAs, 29 x 10^-5^ were snoRNAs, and 99.35% were others. Of the total small RNA reads from male *Varroa*, 25 x 10^-4^% were miRNAs, 15 x 10^-3^ were rRNAs, 94 x 10^-3^ were tRNAs, 4 x 10^-4^ were snRNAs, 2.35 x 10^-5^ were snoRNAs, and 99.89% were others.

### Prediction of Target Genes and Gene Ontology (GO) and Functional Enrichment Analyses in the Target Network

**Figure 6:**
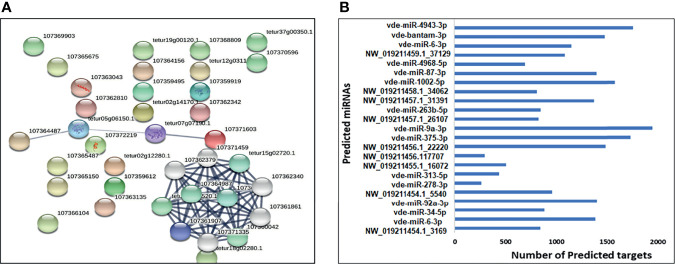
**(A)** A network built exclusively from *Varroa* proteins targeted by predicted upregulated/downregulated miRNAs. **(B)** The total number of predicted target transcripts significantly upregulated and downregulated in female *Varroa* miRNAs relative to male miRNAs.

STRING web analysis (**Figure 6A**) showed that target proteins for the 31 predicted miRNAs (18 upregulated and 13 downregulated) had more interactions than expected for a random set of proteins of similar size sampled from the *V*. *destructor* genome (number of nodes = 42, number of edges = 97, average node degree = 4.62, average local clustering coefficient = 0.476, expected number of edges = 25, PPI enrichment *p*-value < 1.0e-16). Such enrichment indicates that the proteins are at least partially biologically connected as a group. To minimize the number of false-positive targets, we opted only for targets predicted by all three miRNA target programs (TargetSpy, MIRANDA, and PITA). The predicted miRNAs were therefore of high confidence, and the main KEGG pathways involved were oxidative phosphorylation (12 out of 80), endocytosis (4 out of 116), protein processing in the endoplasmic reticulum (2 out of 109), and other metabolic pathways (14 out of 810). Many target genes were predicted for the differentially-expressed miRNAs in the small RNA-seq data (**Figure 6B**) using the miRNAconsTarget program from sRNAtoolbox (Aparicio-Puerta et al., 2019).

### Validation of Predicted miRNAs in Female *Varroa* by qRT-PCR

**Figure 7:**
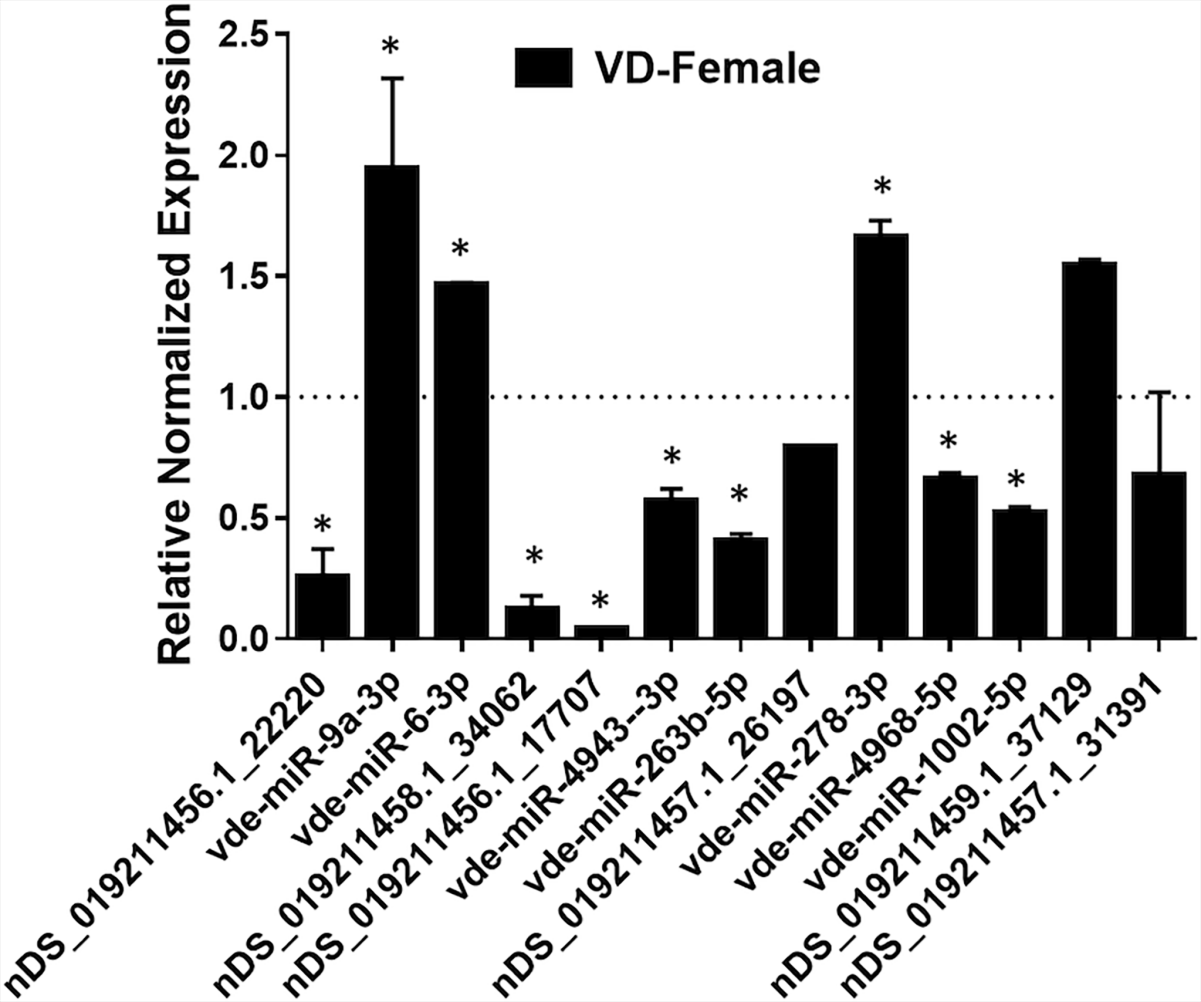
qPCR validation of selected mature miRNAs (high confidence) in female *Varroa* differentially expressed relative to male *Varroa*. Statistical significance for qRT-PCR-based differential expression was determined by the 2-tailed Student’s *t*-test, where * denotes *p*<0.05. miRNA expression of female *Varroa* was normalized to that of male *Varroa* (indicated as 1 on y-axis). Succinate dehydrogenase (*SDHA*) was used as a housekeeping control.

The expression of 13 predicted differentially-expressed miRNAs were validated using qRT-PCR (**Figure 7**). The qRT-PCR results of differentially-expressed miRNAs matched the NGS patterns for the majority of evaluated miRNAs. Inconsistencies in NGS and qRT-PCR data were found for vde-miR-4943-3p, nDS_019211456.1_17707, and vdemiR-4968-5p, which could be due to using different methodologies to quantify miRNA expression (Saldaña et al., 2017). Inconsistencies between NGS and qRT-PCR data have been reported previously (Hermance et al., 2019).

## Discussion

MicroRNAs are indispensable post-transcriptional regulators of gene expression in various biological pathways including cell growth, apoptosis, metamorphosis, development, and vector competence (Kloosterman and Plasterk, 2006). However, little is known about miRNA expression in parasitic mites such as *Varroa destructor*. Therefore, identifying and understanding miRNA expression profiles in male and female *Varroa* expand our fundamental knowledge of mite miRNAs and provide insight into the development and vector competence of *Varroa*.

A conservative *in silico* approach identified 306 miRNAs, among which 80 were low-confidence and 50 high-confidence miRNAs. Among these high-confidence miRNAs, 18 were up- and 13 were downregulated in female compared with male *Varroa*. qRT-PCR validation revealed upregulation of several predicted miRNAs including vde-miR-87-3p, vde-bantam-3p, vde-miR-375-3p, and vde-miR-34-5p in DWV-B-infected female *Varroa* samples, which have known roles in DENV replication, CHIKV infection, and immune responses to DENV-2 infection in mosquitoes (Sempere et al., 2003; Hussain et al., 2013; Yan et al., 2014; Liu et al., 2015; Lucas et al., 2015a; Maharaj et al., 2015). This small RNA dataset provides a new resource to characterize miRNA function in mite biology. Other predicted *Varroa* miRNAs have also been implicated in development, pesticide resistance, metamorphosis, immune responses, and reproduction (**Figure 4A** and **Table 2**) in other arthropods (Sempere et al., 2003; Hussain et al., 2013; Yan et al., 2014; Lei et al., 2015; Liu et al., 2015; Lucas et al., 2015a; Maharaj et al., 2015). The new and novel miRNAs predicted in this study will require functional validation using miRNA inhibitory experiments.

Among the predicted miRNAs in this study, many were novel and are likely *Varroa*-specific (**Table S2**). Several were homologous to the miRNAs already identified in other arthropods such as *Apis mellifera*, *Drosophila melanogaster*, and *Ixodes scapularis* (**Tables S2, S4**), suggesting conserved roles for these miRNAs (Marco et al., 2010; Fonseca et al., 2021). One hundred and twelve miRNAs detected in *Varroa* in this study have already been identified in *A. mellifera*. Indeed, it has been shown that siRNA molecules ingested by *A. mellifera* can be transferred to ectoparasitic *Varroa* mites and vice-versa. Small RNA exchange of this kind suggests cross-kingdom interactions and could possibly contribute to the shared miRNA profile (Garbian et al., 2012; Fonseca et al., 2021). In general, the most conserved miRNAs are involved in common cellular, biological, and development processes.

Several of the predicted miRNAs identified in this study are also conserved in *D. melanogaster* such as let-7-5p, bantam-3p, miR-8-3p, miR-34-5p, miR-263a-5p, miR-87-3p, miR-252-5p, miR-12-5p, miR-375-3p, miR-9a-3p, miR-306-5p, miR-133-3p, miR-6-3p, miR-276-a-3p, miR-1002-5p, and miR-304-5p, which are known to have conserved roles in other arthropods as well. bantam-3p targets the proapoptotic gene *hid* and is involved in several cellular processes such as proliferation, apoptosis, development, and the circadian clock (Brennecke et al., 2003; Kadener et al., 2009). In *Aedes aegypti*, bantam-3p is significantly upregulated during pupal developmental, and the highest expression has been reported at the mid-pupal period (Bryant et al., 2010). In another study, bantam-3p was most abundantly expressed in both pupal and adult male and female mosquitoes, indicating its functional importance (Feng et al., 2018) and a possible role in *Varroa* development. Another conserved miRNA, miR-8-3p, was significantly upregulated in *Aedes aegypti* during pupation and has the highest expression in the mid-pupal period. Bryant et al. (2010) showed upregulation of miR-8-3p in the fat body of a blood-fed female mosquito and suggested a potential regulatory role in *Ae. aegypti* reproduction. Different to in *Ae. aegypti*, miR-8-3p is abundantly expressed in *Anopheles stephensi* developmental stages (Feng et al., 2018) and found to be equally expressed in uninfected or infected *Ae. albopictus* saliva upon infection with CHIKV (Maharaj et al., 2015). In *Ae. aegypti*, miR-8-3p has been validated to target SWIM (secreted wingless-interacting molecule), thereby regulating reproductive events (Lucas et al., 2015a). Additionally, miR-8-3p shows cell type-specific expression and it is expressed in S2 cells (a cell line derived from *Drosophila melanogaster* embryos). Its temporal expression is less restricted in *Drosophila* and has been observed across all developmental stages, occasionally with significant variation in expression (Jin et al., 2012). Based on these studies and the conserved nature of miR-8-3p, it is also likely to play a role in *Varroa* development, reproduction, and virus infection and requires further investigation.

Another *Varroa-*predicted miRNA common to *Drosophila* was miR-34-5p, the expression of which is more pronounced in female midguts in *A. gambiae* (Winter et al., 2007). In contrast, miR-34-5p is downregulated in *Drosophila* during metamorphosis (pupa to adult stage transition) (Sempere et al., 2003). Interestingly, in *A. gambiae*, miR-34-5p expression was downregulated in the midgut upon *Plasmodium falciparum* infection (Dennison et al., 2015). miR-34-5p has been suggested to contribute to anti-pathogen and immune responses during DENV-2 infection in *Ae. albopictus* (Liu et al., 2015). Again, the conserved nature of this miRNA suggests a possible role in development regulation in *Varroa*, but this requires further functional validation. qRT-PCR showed ~5000-fold upregulation of miR-34-5p in DWV-B-infected *Varroa* females compared with DWV-B infected males, indicating a possible role for this miRNA in anti-DWV-B and immune responses during DWV-B infection, or perhaps facilitating DWV-B survival inside *Varroa*. Similarly, miR-87-3p was upregulated in female *Varroa*, and previous studies have suggested a role for this miRNA in *Drosophila* development (Sempere et al., 2003) and anti-viral immune responses during DENV-2 infection in *Ae. albopictus* (Liu et al., 2015). Our qRT-PCR analysis also demonstrated ~300-fold upregulation in DWV-B-infected *Varroa* females, suggesting a possible role either in anti-DWV-B immune responses or in harboring DWV-B.

miR-375-3p was another miRNA of interest in our analysis. In *Ae. aegypti*, miR-375-3p is expressed in blood-fed mosquitoes, and miR-375 was found to regulate DENV replication, enhancing DENV-2 infection in an *Ae*. *aegypti* cell line (Hussain et al., 2013). In *Ae. Aegypti*, miR-375 targets include *Cactus* and *REL1*, and the injection of an miRNA mimic into mosquitoes altered the expression of immune gene transcripts, suggesting that aae-miR-375 enhances DENV-2 infection in *Ae*. *aegypti* (Hussain et al., 2013). Our qRT-PCR data revealed ~20,000-fold upregulation in DWV-B-infected *Varroa*, suggesting a possible role in DWV-B infection. Interestingly, miR-263a-5p was reported as upregulated in uninfected and CHIKV-infected *Ae. aegypti* saliva (Maharaj et al., 2015). miR263a-5p is constitutively expressed across many developmental stages in several mosquito species (Hu et al., 2015). Another conserved miRNA, dme-miR-252-5p, was induced over three-fold after DENV-2 infection in the *Ae*. *albopictu*s C6/36 cell line and inhibited DENV replication by suppressing the expression of the DENV envelope (E) protein (Yan et al., 2014). In *An. gambiae*, dme-miR-12-5p was found to be expressed in the thorax of males and females but predominantly in midguts and constitutively expressed in their heads (Winter et al., 2007). dme-miR-12-5p targets DNA replication licensing factor (*MCM6*) and monocarboxylate transporter (*MCT1*) genes, as validated in *Ae. aegypti*, through which it affects *Wolbachia* density in host cells (Osei-Amo et al., 2012). dme-mir-279-3p is expressed evenly and ubiquitously throughout the *An*. *gambiae* body (Winter et al., 2007). dme-miR-278-3p was also predicted in our data, and it is highly and abundantly expressed in several reported studies (Feng et al., 2018). dme-miR-278-3p was upregulated in *Culex pipiens pallens* upon exposure to pyrethroid, a widely and indiscriminately used insecticide, where miR-278-3p targets *CYP6AG11* to regulate pyrethroid resistance (Lei et al., 2015). The pyrethroid tau-fluvalinate (Apistan^®^) was one of the first synthetic varroacides registered in the United States, and pyrethroid-resistant *Varroa* is a major challenge in controlling this mite. Pyrethroid-resistant *Varroa* are associated with point mutations in the voltage-gated sodium channel gene at position 925 (Millán-Leiva et al., 2021). Further dissection of these mechanisms might provide a fruitful means to overcome pyrethroid resistance in *Varroa*.

The miRNAs predicted through *Varroa* small RNA-sequencing allow us to generate a list of conserved miRNA targets that may be involved in regulating key biological processes. As discussed earlier, conserved homologous *Drosophila* miRNAs predicted in *Varroa* (**Figure 4A** and **Table 2**) include vde-miR-87-3p, vde-bantam-3p, vde-miR-375-3p, and vde-miR-34-5p. These miRNAs have been shown to play a role in inhibiting or replicating DENV and CHIKV in mosquito species (Sempere et al., 2003; Hussain et al., 2013; Maharaj et al., 2015). These miRNAs were significantly upregulated (100-20,000-fold) in DWV-B-infected *Varroa* females (**Figure 4B**), indicating a functional role for these miRNAs in viral load warranting a detailed study to characterize these miRNAs in *Varroa*-borne virus transmission studies. As the expression of vde-miR-87-3p, vde-bantam-3p, vde-miR-375-3p, and vde-miR-34-5p was significantly higher in females than males, it is also possible that upregulation of these miRNAs could be sex- or age-specific rather than being related to DWV-B infection, and this requires further clarification. Indeed, some of the predicted mature miRNAs such as nDS_019211455.1_10989 and nDS_019211455.1_16072 were male specific and three of the predicted miRNAs (nDS_019211455.1_10999, nDS_019211459.1_37116, and nDS_019211455.1_10993; **Tables S1**, **S3**) were female-specific. A previous study in the nematode *Ascaris suum* predicted the role of gender-specific miRNAs as elongation factors, heat shock proteins, and growth factors essential for organism development (Xu et al., 2013). Moreover, sperm proteins and sperm cell motility proteins were targets of male-specific miRNAs, while ovarian message proteins were targets of female-specific miRNAs (Xu et al., 2013). These male- and female-specific miRNAs in *Varroa* need further functional exploration.

**Figure 8:**
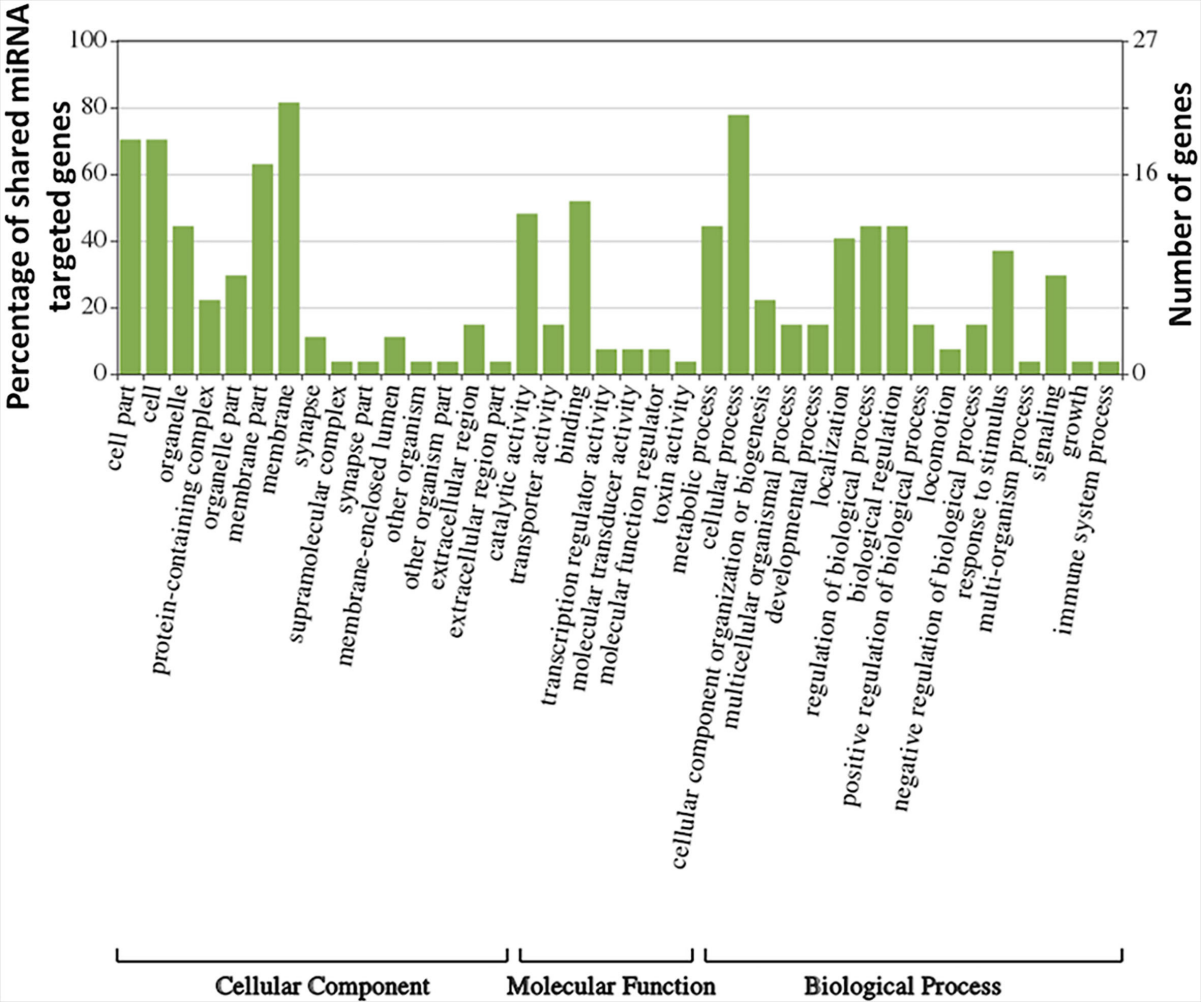
Gene ontology (GO)-derived biological processes related to genes targeted by upregulated/downregulated miRNAs in female *V. destructor*.

In this study, the predominant peak of identified miRNAs corresponded to 24 nucleotides, consistent with previous genome-wide miRNA identification studies in *Varroa* (Fonseca et al., 2021). These miRNAs are major contributors to the total small RNA complement in male and female *Varroa*. It has been suggested that miRNAs might be actively involved in vector competence of *Varroa*-borne viruses. Using publicly-available *Varroa* genome data, we annotated and evaluated possible targets and functions of putative female *Varroa* miRNAs showing *in silico* upregulation or downregulation relative to males. The most conserved miRNAs were involved in several biological, cellular, and developmental processes. Genes controlled by miRNAs conserved in *D. melanogaster* and *Varroa* identified in this study regulate development, metamorphosis, proliferation, apoptosis, reproduction (*Aedes aegypti*), insecticide resistance, and also antiviral immune responses (**Table 2**). Interestingly, analysis of GO terms related to genes regulated by the shared miRNAs revealed many specific processes known to be regulated by the same miRNAs in *D. melanogaster* such as developmental processes, metamorphosis, and immune responses, reinforcing their conserved role (**Figure 8** and **Table 2**). The main KEGG pathways predicted by STRING web analysis were oxidative phosphorylation, endocytosis, protein processing in the endoplasmic reticulum (ER), and other metabolic pathways (**Table 3**). Proteins involved in endocytosis and protein processing in the ER could provide significant clues about viral infection (Wang et al., 2013; Rosche et al., 2021) in *Varroa*.

**Table 3:** Functional enrichment in predicted protein network (STRING).

Local Network Cluster (STRING)				
*Cluster*	*Description*	*Count in network*	*Strength*	*False discovery rate*
CL-6642	mixed, incl. zinc-finger domain, and ETC complex I subunit consensus	6 of 8	2.57	1.90e-12
CL-6638	Oxidative phosphorylation	8 of 13	2.48	7.16e-16
CL-6668	mixed, incl. Translocase, and 4Fe-4S binding domain	3 of 6	2.39	8.24e-06
CL-6636	Oxidative phosphorylation	11 of 25	2.34	3.76e-20
CL-6630	Oxidative phosphorylation and porin, eukaryotic type	12 of 67	1.95	2.16e-18
CL-8320	mixed, incl. Protein transport, and Pleckstrin homology domain.	3 of 148	1	3.83e-02
				
**KEGG Pathways**				
*Pathway*	*Description*	*Count in network*	*strength*	*False discovery rate*
tut00190	Oxidative phosphorylation	10 of 80	1.79	1.11e-14
tut04144	Endocytosis	3 0f 116	1.11	0.0042
tut01100	Metabolic pathways	12 of 810	0.86	1.46e-07
**Annotated Keywords (Uniprot)**				
*Keyword*	*Description*	*Count in network*	*Strength*	*False discovery rate*
KW-0342	GTP-binding	3 of 74	1.3	0.007
KW-0813	Transport	5 of 358	0.84	0.0197
**Protein Domains (Pfam)**				
*Domain*	*Description*	*Count in network*	*Strength*	*False discovery rate*
PF05347	Complex 1 protein (LYR family)	2 of 6	2.22	0.007
PF00350	Dynamin family	2 of 8	2.09	0.007
PF00153	Mitochondrial carrier protein	2 of 40	1.39	0.0343
PF00520	Ion transport protein	2 of 43	1.36	0.0343
PF08477	Ras of Complex, Roc. Domain of DAPkinase	3 of 71	1.32	0.0091
PF00025	ADP-ribosylation factor family	3 of 70	1.32	0.0091
PF00071	Ras family	3 of 79	1.27	0.0091
**Protein Domains and Features (InterPro)**				
*Domain*	*Description*	*Count in network*	*Strength*	*False discovery rate*
IPR030381	Dynamin-type guanine nucleotide-binding (G) domain	2 of 5	2.29	0.0098
IPR008011	Complex 1 LYR protein	2 of 6	2.22	0.0098
IPR022812	Dynamin superfamily	2 of 7	2.15	0.0098
IPR020849	Small GTPase superfamily, Ras-type	2 of 18	1.74	0.0145
IPR002067	Mitochondrial carrier protein	2 of 18	1.74	0.0145
IPR005821	Ion transport domain	2 of 31	1.5	0.0264
IPR023395	Mitochondrial carrier domain superfamily	2 of 36	1.44	0.0314
IPR018108	Mitochondrial substrate/solute carrier	2 of 36	1.44	0.0314
IPR001806	Small GTPase	3 of 60	1.39	0.0098
IPR005225	Small GTP-binding protein domain	3 of 79	1.27	0.0142
IPR027417	P-loop containing nucleoside triphosphate hydrolase	6 of 557	0.72	0.0145
**Protein Domains (SMART)**				
*Domain*	*Description*	*Count in network*	*Strength*	*False discovery rate*
SM00173	Ras subfamily of RAS small GTPases	2 of 8	2.09	0.0034

## Conclusions

This study provides several potential miRNA targets that now require functional validation to confirm their role in the pathophysiology of *Varroa*. Characterization of *Varroa* miRNAs paves the way for a deeper understanding of *Varroa* biology and survival mechanisms of DWV inside *Varroa* so that these processes can be targeted to control and prevent both mites and the vectors they transmit.

## Data Availability Statement

The datasets presented in this study can be found in online repositories. The names of the repository/repositories and accession number(s) can be found below: NCBI (accession: PRJNA794145).

## Author Contributions

Conceptualization: DK and SK. Data curation: DK. Formal analysis: DK. Funding acquisition: SK and JA. Investigation: DK, MA, FT, MG, JA, and SK. Methodology: DK and SK. Project administration: SK. Resources: JA and SK. Supervision; SK. Validation: DK and SK. Visualization: DK. Writing, original draft: DK and SK. Writing, review & editing: DK, MA, MG, JA, and SK. All authors contributed to the article and approved the submitted version.

## Funding

This research was funded by a USDA ARS cooperative agreement and the Mississippi INBRE (an institutional Award (IDeA) from the National Institute of General Medical Sciences of the National Institutes of Health under award P20GM103476).

## Conflict of Interest

The authors declare that the research was conducted in the absence of any commercial or financial relationships that could be construed as a potential conflict of interest.

## Publisher’s Note

All claims expressed in this article are solely those of the authors and do not necessarily represent those of their affiliated organizations, or those of the publisher, the editors and the reviewers. Any product that may be evaluated in this article, or claim that may be made by its manufacturer, is not guaranteed or endorsed by the publisher.
