# Supplementary material for: An Insight Into the microRNA Profile of the Ectoparasitic Mite Varroa destructor (Acari: Varroidae), the Primary Vector of Honey Bee Deformed Wing Virus
Source: Front Cell Infect Microbiol. 2022 Mar 16;12:847000. doi: 10.3389/fcimb.2022.847000 (PMC8966896; doi:10.3389/fcimb.2022.847000)
Supplement: Supplementary file 1 [file DataSheet_1.docx]

Supplementary Material

# Supplementary Tables Table S1. qRT-PCR primers used in this study

| **miRNA / gene** | **Forward** | **Reverse** |
| --- | --- | --- |
| vde-bantam-3p | cgcagtgagatcattgtgaaag | caggtccagtttttttttttttttaatc |
| vde-miR-375-3p | agtttgttcgttcggctc | ggtccagtttttttttttttttgac |
| vde-miR-34-5p | cagtggcagtgaggttag | ccagtttttttttttttttcaaccag |
| vde-miR-6-3p | tcacagccacctttgatg | ccagtttttttttttttttaggctc |
| nDS_019211459.1_37129 | cgcgtcattgagaactttc | ggtccagtttttttttttttttggt |
| nDS_019211457.1_31391 | cgcagcaagttaatctctaag | ggtccagtttttttttttttttattgg |
| nDS_019211456.1_22220 | caggtgtgcgtgagataatc | caggtccagtttttttttttttttatttag |
| vde-miR-263b-5p | cagtttggcactagcaca | caggtccagtttttttttttttttaca |
| vde-miR-9a-3p | gcgcagtaaagctataagact | ggtccagtttttttttttttttgac |
| nDS_019211457.1_26197 | tcagatctacccgttccag | gtccagtttttttttttttttcgag |
| vde-miR-278-3p | ggtgggatcttcgtcca | caggtccagtttttttttttttttaac |
| vde-miR-87-3p | ggtgagcaaagtttcaggt | ggtccagtttttttttttttttacaca |
| nDS_019211458.1_34062 | cgcagttgtgaccgattc | agtttttttttttttttgcccattg |
| nDS_019211456.1_17707 | cagtcaccgggttgtca | caggtccagtttttttttttttttaga |
| vde-miR-4943-3p | gtgaacacagcttgtggt | ggtccagtttttttttttttttactga |
| Succinate dehydrogenase (SDHA, 108 bps), housekeeping gene | aaaccgggaacgaccttatc | tccaatccttccaactgtcc |

**Table S2.** *Varroa destructor* (VD) microRNA annotation (hivdegh confidence, medium confidence, low confidence) based on standard criteria.

| **Novel miRNAs predicted by miRDeep2** | **miRDeep2 score** | **Total read count** | **Mature read count** | **miRNA with same seed** | **Consensus mature sequence** | **Consensus star sequence** |
| --- | --- | --- | --- | --- | --- | --- |
| provisional id |  |  |  |  |  |  |
| vde-bantam-3p High Confidence | 4800000 | 1E+07 | 1E+07 | dme-bantam-3p | ugagaucauugugaaagcugauu | cgggcuuucucacugauuuccaga |
| nDS_019211454.1_2001 High Confidence | 790000 | 2E+06 | 2E+06 |  | uacuuugagucgguacgaauccu | gguuuguaccagcucucaguuagu |
| vde-miR-8-3p High Confidence | 640000 | 1E+06 | 1E+06 | dme-miR-8-3p | uaauacugucagguaaagauguc | caucuuacuugauagcguuaga |
| vde-miR-276a-3p High Confidence | 550000 | 1E+06 | 1E+06 | dme-miR-276a-3p | uaggaacuucaaaccaugcucg | cgcaugguuugagggucgugcc |
| nDs_019211454.1_5072 High Confidence | 340000 | 7E+05 | 644114 |  | ucucacuagccugucuuugacg | ugaaagacaaggguagugagaug |
| vde-miR-993-5p High Confidence | 310000 | 6E+05 | 623629 | dme-miR-993-5p | uacccuguugauccgaaucugu | caaauugggaucacgggggguc |
| nDs_019211460.1_41606 High Confidence | 310000 | 6E+05 | 522521 |  | ucuuugguuaucuagcuguauga | auaaagcuagguuaccaaaguua |
| vde-miR-4943-3p High Confidence | 280000 | 6E+05 | 552545 | dme-miR-4943-3p | ugaacacagcuugugguaaucagu | ugauuaccacucuggguucguac |
| vde-miR-277-3p low Confidence | 190000 | 4E+05 | 385737 | dme-miR-277-3p | uaaaugcaugcucugaaaugga | cguuccagagaugcuuuugca |
| vde-miR-279-3p low Confidence | 170000 | 3E+05 | 330324 | dme-miR-279-3p | ugacuagaucaacacucaucu | gaugagugucgguuuggugaaug |
| nDs_019211456.1_17707 High Confidence | 150000 | 3E+05 | 302162 |  | ucaccggguugucaugcaauucu | auugcauguuaacccgggggac |
| vde-miR-305-5p low Confidence | 130000 | 3E+05 | 270441 | dme-miR-305-5p | auuguacuucaucaggugcucu | aggcaucucauggagugcaaaug |
| dme-miR-279-3p High Confidence | 120000 | 2E+05 | 234733 | dme-miR-279-3p | ugacuagauuuacacucaucca | gaugaguguaugccuagugcauu |
| nDs_019211458.1_34062 High Confidence | 110000 | 2E+05 | 229144 |  | uugugaccgauucaaugggca | cccauugaauuguucacuacu |
| vde-miR-12-5p High Confidence | 110000 | 2E+05 | 222621 | dme-miR-12-5p | ugaguauuacaucagguacuggu | caguaccgaugucauauucg |
| vde-miR-12-5p High Confidence | 110000 | 2E+05 | 219832 | dme-miR-12-5p | ugaguauuacaucagguacuggu | caguaucgaugucauauucuc |
| nDs_019211455.1_12076 High Confidence | 89000 | 2E+05 | 175252 |  | uugcauagucacaaaagugaug | ccacuuuugugauuggugcaauu |
| nDs_019211457.1_31182 | 89000 | 2E+05 | 175252 |  | uugcauagucacaaaagugaug | ucacuuuugugauuggugcaguu |
| vde-miR-87-3p High Confidence | 76000 | 2E+05 | 147378 | dme-miR-87-3p | gugagcaaaguuucaggugugu | cggccugaugccuugucucaaccu |
| nDs_019211458.1_35970 High Confidence | 75000 | 1E+05 | 147378 |  | gugagcaaaguuucaggugugu | agccugaagcguugucucaaccu |
| vde-miR-263a-5p High Confidence | 71000 | 1E+05 | 139069 | dme-miR-263a-5p | aauggcacugcaagaauucacgg | gugcguucugcagugcccucug |
| vde-miR-306-5p High Confidence | 68000 | 1E+05 | 131371 | dme-miR-306-5p | ucagguaccugaaguagcgcgc | cgugcuacggcaggugcuuguggcu |
| nDs_019211455.1_13802 High Confidence | 65000 | 1E+05 | 115260 |  | caaguucgaauuuucggguagc | uacccguacauccgaacuugu |
| vde-miR-6-3p High Confidence | 63000 | 1E+05 | 105151 | dme-miR-6-3p | uaucacagccagcuuugaugagg | ucaccaaaguggcugugcagacg |
| vde-miR-278-3p High Confidence | 63000 | 1E+05 | 118829 | dme-miR-278-3p | ucggugggaucuucguccaguu | cgggacgggauucaaacggacc |
| nDs_019211454.1_5076 High Confidence | 60000 | 1E+05 | 73170 |  | uaucacagccaucuuugaugacu | ucaucgaguuggcuguggucu |
| vde-miR-9a-3p High Confidence | 56000 | 1E+05 | 84660 | dme-miR-9a-3p | uaaagcuauaagacugcgguc | cccguagucuuguggcuuugg |
| nDs_019211457.1_26197 High Confidence | 52000 | 1E+05 | 99626 |  | ucagaucuacccguuccagcucg | aguuggaauguagcgcuggca |
| nDs_019211459.1_37127 Confidence low | 51000 | 1E+05 | 101675 |  | ucaccgguuugaauucgucgaa | cgccgaauuuguccgcggug |
| vde-miR-87-3p | 49000 | 96636 | 78876 | dme-miR-87-3p | gugagcaagguuucaggugugu | cggccuggaguguugcucaucc |
| vde-miR-279-3p High Confidence | 46000 | 90833 | 90355 | dme-miR-279-3p | ugacuagaacaacacucacgcgg | cgugaauguuuuucuggugcaug |
| nDs_019211454.1_21 High Confidence | 46000 | 90557 | 90164 |  | ugacuagaaccucacucacgccg | gugugaguggguucuuguugug |
| nDs_019211454.1_7299 | 39000 | 78378 | 78316 |  | aucccggacgagcccgca | gcgagaggucccggguuc |
| vde-miR-34-5p High Confidence | 39000 | 76669 | 74994 | dme-miR-34-5p | uggcagugugguuagcugguugu | aaucaguaacugcacugccucu |
| vde-let-87 High Confidence | 38000 | 76151 | 73985 | dme-let-87 | cgagguaguagguuguauaguu | cuauacauccaacuagcucaugc |
| vde-miR-1002-5p High Confidence | 37000 | 73757 | 72973 | dme-miR-1002-5p | uuaaguaguagugccgcaagua | ccugcgcauuacugccuaauc |
| vde-miR-133-3p High Confidence | 37000 | 73652 | 73575 | dme-miR-133-3p | uugguccccuucaaccagcugu | agcuguuugggggagcaccaauu |
| nDs_019211455.1_10989 High Confidence  Male-Specific | 36000 |  | 70766 |  | augacuagaaccucacucacggg | cgugaguggauuucuggugcauu |
| vde-miR-375-3p High Confidence | 33000 | 65935 | 64736 | dme-miR-375-3p | uuuguucguucggcucgaguca | ccuugaucccuaugaacaaaac |
| vde-miR-34-5p High Confidence | 31000 | 61397 | 51077 | dme-miR-34-5p | uggcagugagguuagcugguug | aucggcgaccuuggcugcccgg |
| vde-miR-6-3p High Confidence | 30000 | 59093 | 57757 | dme-miR-6-3p | caucacagccaccuuugaugagccu | gccaucaagugguugugaagug |
| vde-miR-252-5p High Confidence | 28000 | 56853 | 39151 | dme-miR-252-5p | cuaaguacuggcgccguaggagu | uccugcgcgcuguugugcuuacc |
| nDs_019211455.1_16072 High Confidence  Male-Specific | 28000 | 56719 | 52601 |  | caaugcccaaggaaaccccgaa | cgggcguuccucggcgcauuuuc |
| vde-miR-92a-3p Confidence low | 28000 | 55456 | 55391 | dme-miR-92a-3p | uauugcacauguauccggccuu | gguuggauucgugugccaauguu |
| vde-miR-4968-5p High Confidence | 26000 | 51761 | 50159 | dme-miR-4968-5p | uagcugcccagugaagggcugu | cguucuucgcggggucagcuagc |
| nDs_019211458.1_31630 High Confidence | 23000 | 46340 | 39942 |  | uggcugaacauuguuaugcgug | cgcgucgcaaugcucgguuagc |
| nDs_019211457.1_26374 Confidence Low | 22000 | 44994 | 44958 |  | uuuugauuguugcucagaaggcg | accuuuuagcaacaaucaaauu |
| vde-miR-92a-3p Confidence Low | 22000 | 43801 | 43507 | dme-miR-92a-3p | uauugcacucguuugggccuua | cgguccaaacgggcgucaaucuu |
| nDs_019211455.1_9741 High Confidence | 21000 | 41373 | 34120 |  | uuuguucguucggcucgaguua | acuugauccgggcgaaacaaagc |
| nDs_019211454.1_5540 High Confidence | 19000 | 38298 | 36815 |  | uauacguccgaagcacugagag | cuuggugcuccggaucguaugua |
| vde-miR-304-5p High Confidence | 17000 | 34387 | 29412 | dme-miR-304-5p | uaaucucauagguaucucuggga | ccggagacgccugcugagguuagu |
| nDs_019211456.1_22220 High Confidence | 15000 | 31030 | 26238 |  | gugugcgugagauaaucuaaau | cgggauuaucccacgagcaagc |
| nDs_019211457.1_31391 High Confidence | 13000 | 26714 | 23889 |  | caaguuaaucucuaagcccaau | ugggcuugaagauuagcuuaga |
| vde-miR-92a-3p Confidence Low | 13000 | 25572 | 25175 | dme-miR-92a-3p | uauugcacccgucccggccuc | gguuuuuggaugggugacaauauu |
| nDs_019211459.1_37129 High Confidence | 11000 | 22697 | 18618 |  | cgcgucauugagaacuuucacc | ugaaaguucccuaugaugcaguu |
| nDs_019211459.1_37113 Confidence Low | 10000 | 21118 | 21099 |  | ucaccggguuaaauuuagucuga | cgacgaauuuagccacgguagcc |
| nDs_019211456.1_22222 Confidence Low | 10000 | 20016 | 19550 |  | gugugcgugagauaauccuaau | ugggauuacucauugcacaagccg |
| nDs_019211459.1_37125 Confidence Low | 9900 | 19566 | 19548 |  | ucaccgggugagauucgucgaa | ccgaaaaaucucucagcgguaac |
| nDs_019211456.1_22218 High Confidence | 9400 | 18535 | 17714 |  | gugugcgugguucaaucccuau | agggguuguccauugcacaagc |
| nDs_019211455.1_10999 High Confidence  Female-Specific | 8800 | 17321 | 16489 |  | ucaccgggcggacauuugcgcg | cgugaauguuuacucuggucaug |
| nDs_019211460.1_45886 Confidence Low | 8500 | 16703 | 16676 |  | uguagucucgaagaaacgucggu | cggcguuuuucuaggcuucgca |
| vde-miR-958-3p Confidence Low | 8400 | 16654 | 9066 | dme-miR-958-3p | ugagauucaacuucucuauuugu | aaguagagaggucgaaucuucc |
| vde-miR-263b-5p High Confidence | 8000 | 15747 | 14220 | dme-miR-263b-5p | uuuggcacuagcacauuuuugu | aaaauugugccggugucaacu |
| NW_019211454.1_3970 Confidence Low | 7700 | 15234 | 14095 |  | agaaagaccuuucagcucgagg | ucgugcuggaaaguccucccca |
| vde-miR-993-3p Confidence Low | 7500 | 14754 | 14587 | dme-miR-993-3p | gaagcucguaucuacagauaucu | auagccuguagacacgggcuaacu |
| vde-miR-1-3p High Confidence | 7100 | 13925 | 9266 | dme-miR-1-3p | uggaauguaaagaaguauggag | caauacuucuuugccaucccaua |
| vde-miR-981-3p Confidence Low | 6100 | 12041 | 11992 | dme-miR-981-3p | uucguugucguagaaaccugag | gagguuucuugauaagcgagca |
| nDs_019212411.1_47808  Confidence medium | 3900 | 7656 | 5782 |  | caaagagaacucuuuuaaagga | cuuuaaaagguuuuucuuugcu |
| vde-miR-125-5p | 3600 | 7176 | 6576 | dme-miR-125-5p | ucccugagauccuuacuugugg | ccgggcaaggacucucgggcgu |
| vde-miR-124-3p Confidence Low | 3400 | 6730 | 6636 | dme-miR-124-3p | uaaggcacgcggugaaugcca | ggcgugcacuggguguccuuaug |
| vde -miR-219-5p Confidence Low | 3100 | 6139 | 5674 | dme-miR-219-5p | ugauuguccaaacgcaauucuug | agaacugugaguggacaucauu |
| nDs_019211460.1_43831 Confidence Low | 2200 | 4458 | 4424 |  | auuccuucgcuggcagaaacgu | gguucugcccguggaggaggugc |
| nDs_019211459.1_37116 Confidence Low  Female-specific | 2000 | 4046 | 3939 |  | ugaaaguuuccuuugaugcggu | cgcgucauuggaagcuuccacc |
| vde-miR-124-3p Confidence Low | 2000 | 3988 | 3969 | dme-miR-124-3p | uaaggcacuuccaucagagaag | ccucugaucgaguggccaugcc |
| nDs_019211457.1_31140 Confidence Low | 1300 | 2644 | 1486 |  | aacuauucuccaucaggcccgu | uggcaagaugguggcauaguug |
| vde-miR-998-3p | 1200 | 2543 | 2460 | dme-miR-998-3p | uagcaccauuugaauucaguuc | acuggguucuucuggugaauaga |
| nDs_019211457.1_28709 | 1100 | 2288 | 2286 |  | acgaaguagauaugaauggcaca | gcgccauccauaucuacuucguuc |
| vde-miR-7-5p | 1100 | 2176 | 1799 | dme-miR-7-5p | uggaagacuugugauuuuguuguu | caauaaaucacugucuucuuu |
| nDs_019211454.1_3169 Confidence medium | 1100 | 2182 | 2093 |  | cuauccugcagaaagcacaccgga | cgggacuuucugcaggaagacu |
| nDs_019211457.1_31299 Confidence medium | 990 | 1952 | 1937 |  | ucugacugcugccuacacgugu | cacgucgagguggcagcagcag |
| nDs_019211455.1_10993Confidence medium  Female-specific | 980 | 1934 | 1774 |  | ucaccgggucacaaccaucucg | aggagguuggggcucugguggug |
| nDs_019211455.1_10997 | 910 | 1798 | 1614 |  | ucaccggguagauauaugcgcg | ccgcaagugucuacuuugguaaug |
| nDs_019211460.1_43431 Confidence medium | 910 | 1786 | 1691 |  | uaugacgucgauguguggcgau | cgucagacgucgucgucauuaug |
| vde-miR-313-5p Confidence medium | 840 | 1653 | 1077 | dme-miR-313-5p | ugcugcggccgaagaaccucugu | ugagguugucugaaguagcagu |
| nDs_019211455.1_10995 Confidence medium | 800 | 1578 | 741 |  | ucaccggguaaugauucgugcg | cacgaguaauuacccuguucau |
| nDs_019211455.1_9525 Confidence Low | 760 | 1500 | 1466 |  | uuauugcuugagaauacacgu | guguguucucaagagaauaaca |
| nDs_019211459.1_37119 Confidence Low | 750 | 1479 | 1368 |  | ucaccggguuauauucguucgagc | cggacgauguuaucaacggucacu |
| nDs_019211455.1_11001 Confidence Low | 630 | 1239 | 1212 |  | ucaccgggucagcauuugcgcgu | ccguaaauguccacccugguuaug |
| nDs_019211459.1_37544 Confidence Low | 550 | 1080 | 878 |  | uacguauacugaagguauaccu | agguuacacacaguauacguuc |
| nDs_019211454.1_3053 Confidence Low | 520 | 1028 | 1003 |  | cucuuuaccgcgaucaucucgg | ggguugcgcgcggcaaaguauggc |
| nDs_019211454.1_3252 Confidence medium | 430 | 850 | 793 |  | aaacuuaacugacuggaaacu | uuuccaguaaguuaaguuuucc |
| nDs_019211454.1_5712 Confidence medium | 410 | 808 | 569 |  | uuauaggcgucaugguaugauc | ucguaccgugacgccuauaagu |
| nDs_019211459.1_37418 Confidence Low | 380 | 760 | 265 |  | cgaaaaauucagcagauauauc | uauaucugcugaauuuuucggu |
| nDs_019211459.1_39827 Confidence medium | 370 | 736 | 263 |  | cgaaaaauucagcagauauauc | uauaucugcugaauuuuucggu |
| nDs_019211456.1_16270 Confidence Very Low | 360 | 715 | 449 |  | ucgcauagguuaacagcaccga | cggugcuguuaaccuaugcgag |
| nDs_019211456.1_20739 Confidence Very Low | 350 | 702 | 436 |  | ucgcauagguuaacagcaccga | cggugcuguuaaccuaugcgag |
| nDs_019211454.1_5953 Confidence Very Low | 330 | 661 | 644 |  | uggacuaaaccuggagaggacu | guucgcccaccuuuggucacaua |
| nDs_019211459.1_37097 Confidence Low | 330 | 655 | 584 |  | uuccccugaacauucaccauuu | auggugaacguacaggggaacu |
| nDs_019211457.1_26088 Confidence medium | 330 | 652 | 433 |  | ucaaugacaaccaagaggacau | guccccuugguugucauugagc |
| nDs_019211459.1_39881 Confidence Very Low | 300 | 603 | 601 |  | uuuucgucagugcuugaaggccu | ucuccgagcacuggcaaagug |
| nDs_019211459.1_37117 Confidence Low | 290 | 586 | 558 |  | ucacaggguaagauccgucgga | cgagggaucuuuucacgguuacc |
| nDs_019211454.1_7181 Confidence medium | 260 | 516 | 441 |  | aaacuuaacuuacuggaaacuu | uuuccagucaguuaaguuuucc |
| nDs_019211459.1_41377 Confidence Very Low | 240 | 489 | 473 |  | uggcgcuugucggcugguugauu | ucagccagugggcagcuccuaau |
| nDs_019211457.1_27766 Confidence Very Low | 230 | 467 | 420 |  | uaacguacacucggauacagau | ucuguauccgaguguacguuagu |
| nDs_019211457.1_30953 | 230 | 467 | 420 |  | uaacguacacucggauacagau | ucuguauccgaguguacguuagu |
| nDs_019211454.1_334 | 190 | 378 | 377 |  | aguaaauaaccgguuucaacuc | uugaaaccgguuauuuacug |
| nDs_019211459.1_38286 | 170 | 350 | 345 |  | cguucguuacaccaucgcugcu | gcgccaugcuguaacguccuagu |
| nDs_019211454.1_3684 | 150 | 300 | 153 |  | uagucauggauauggaucaca | gugauccauauccaugacuacu |
| nDs_019211454.1_5866 | 130 | 258 | 229 |  | ucugaucaagaaauaucuaccu | auagauauuuguuggucaggcu |
| nDs_019211459.1_38873 Confidence Very Low | 120 | 249 | 246 |  | cauuaugcgacgguucgcucuau | gggacgaaccguccauaagugcgu |
| nDs_019211455.1_9267 | 110 | 223 | 222 |  | gauuuggaacccggcugcuugc | gaggcagccgggauccaaaucu |
| nDs_019211454.1_7721 | 100 | 199 | 188 |  | aaggauugcgguuaaaguuaca | guaacuuuaaccgcaauccuuacc |
| nDs_019211459.1_38565 Confidence Very Low | 100 | 197 | 167 |  | gguuccguugucguaguugguu | cgaguacgacaacggaaucagu |
| nDs_019211460.1_45536 Confidence Low | 100 | 195 | 173 |  | auggccagucgagacgagaaau | uucucgucuaggcuggccaauau |
| nDs_019211456.1_23856 Confidence Low | 97 | 189 | 176 |  | cugcugccgcaauucccuugcu | cgagggaauugcggcagccgcg |
| nDs_019211458.1_33802 | 82 | 159 | 112 |  | uggggccugucggaauccacu | gcggauuccgacaggccccagc |
| nDs_019211455.1_12486 Confidence Low | 82 | 159 | 104 |  | uuuggcaauaguggacccgauu | cgggagucuacuauugccaaagc |
| nDs_019211456.1_23806 | 78 | 162 | 153 |  | ccaccuagcagcuguaggcgcucg | cggcaacuacucucugcccgggcc |
| nDs_019211455.1_8514 Confidence Low | 77 | 149 | 74 |  | uguugaucugucuaucuauau | auagauagacagaucgacaggc |
| nDs_019211456.1_19039 | 73 | 141 | 139 |  | cuaggcaccacgggcuugucuc | ugacaagcccguggugacuaga |
| nDs_019211460.1_42775 | 71 | 139 | 92 |  | cuagaacuacuuccgucguuuu | aacaacugaaguaguucuggcucu |
| nDs_019211459.1_37154 | 67 | 130 | 86 |  | ggcguaguaaccgaacaaaagga | uuuuguucgguuacuacaaaua |
| nDs_019211457.1_30486 | 58 | 114 | 86 |  | ccaggcgaaauucugucuacgu | ugcagacagaauuuagcccuuagca |
| nDs_019211454.1_3563 | 56 | 108 | 105 |  | uugucgcuggcugucuguugcu | gcucagucagcgagcgauaac |
| nDs_019211458.1_35367 | 53 | 102 | 76 |  | uuuguuuuugagucgcggcuc | gccgcgacucaaaaacaaaua |
| nDs_019211454.1_2946 | 50 | 97 | 92 |  | uaggaguagggcauaaaagaca | ucuuuuaugcccuacuccuau |
| nDs_019211454.1_6023 | 50 | 97 | 94 |  | uugaauuucgucaccuaugcu | uagacgacgaaacucaaaaacu |
| nDs_019211456.1_24314 Very Low confidence | 46 | 93 | 88 |  | uuuggguguauguguauguaga | ugcugcaccuacucaccacugu |
| nDs_019211456.1_19461 Very Low confidence | 45 | 87 | 83 |  | cgcggcugccgcaauucccucg | aagggaauugcggcagcagcg |
| nDs_019211454.1_5895 | 45 | 88 | 86 |  | uuaccucugaaacucuaaaacu | uuuuagaguuucagagguaaua |
| nDs_019211455.1_10717 | 42 | 83 | 77 |  | ucugaaugaagaucuggcaccu | agccagaucuucauucaga |
| nDs_019211455.1_15247 | 38 | 73 | 52 |  | ugccgcucuaggguucucuguccu | gacagagaacccuagagcggca |
| nDs_019211455.1_11112 Very Low confidence | 37 | 70 | 50 |  | ugccgcucuaggguucucuguccu | gacagagaacccuagagcggca |
| nDs_019211454.1_1632 Very Low confidence | 36 | 69 | 61 |  | auaccaugacgccuauaaguu | cuuauaggcgucacgguacga |
| nDs_019211459.1_38759 | 35 | 79 | 64 |  | uagaacauauguagacacggua | ucguugcuacaacuguucucgu |
| nDs_019211454.1_5707 | 34 | 67 | 28 |  | acgugcgggccacaggguacugc | auuauccucaguccgcaucgaau |
| nDs_019211457.1_31021 | 34 | 64 | 57 |  | auagaagcuucgacgacgggcc | cccgucgucgaagcuucu |
| nDs_019211457.1_27818 | 33 | 64 | 57 |  | auagaagcuucgacgacgggcc | cccgucgucgaagcuucu |
| nDs_019211458.1_31502 | 33 | 63 | 23 |  | gccuacaucguuuggauauuccca | gggaauauccaaacgauguagaca |
| nDs_019211454.1_5886 Very Low confidence | 33 | 63 | 56 |  | caagaggacguaaccgugaga | auacgguuacguccccuugugc |
| nDs_019211459.1_40979 | 30 | 58 | 57 |  | cgcaguguuguagaagacaucg | gugucuucuacaacacugccua |
| nDs_019211458.1_34317 | 29 | 57 | 45 |  | aagaucgggaagauggcgccu | acgcccuuuucuaucgacucuu |
| nDs_019211459.1_37870 | 27 | 51 | 49 |  | agcgacuguggcgccaacaggc | cuguuggcgccacagucaucgu |
| nDs_019211456.1_20481 | 25 | 49 | 47 |  | uguuuaccuauguucucgagacu | ccucgaggacacggucaacc |
| nDs_019211458.1_35138 | 25 | 48 | 44 |  | ccaacaaaaguagcguuugguc | ccgaccgccacguuuguugucugacu |
| nDs_019211455.1_10618 Very Low confidence | 24 | 46 | 38 |  | agcgcuuguucggacgucaagc | uugacguacgaacaagcgcuuc |
| nDs_019211455.1_11821 Very Low confidence | 21 | 40 | 38 |  | agcagauaucucuguucguccu | acgaacagaaauaucugcuauu |
| vde-miR-6-3p | 21 | 33 | 17 | dme-miR-6-3p | aaucacagcauuagaauucucuu | gaaaugucaaugcugugaucacc |
| nDs _019211457.1_26482 | 20 | 39 | 22 |  | ucuaauugguccauuuuugac | ucgaaaacggaccaaucacaaau |
| nDs_019211458.1_35363 | 20 | 38 | 34 |  | cgauucggugcuucucguccgc | gacgagaagcaucgaauagaccg |
| nDs_019211458.1_35111 Very Low confidence | 19 | 35 | 30 |  | ucgccaucguuguugcugucgu | aacagcagcggcgauggcg |
| nDs_019211454.1_5759 | 18 | 34 | 33 |  | uccgggucaagaagaucgcacg | ugcgaucuuccugacccuccuc |
| nDs_019211458.1_32741 Very Low confidence | 18 | 36 | 29 |  | uugcgaagcaguaucauguagu | agcuugauacuguuuugcaagu |
| nDs_019211459.1_40365 Very Low confidence | 17 | 33 | 17 |  | uuaucgauauauaauaacgaac | ucguuauuauauaucgauaaug |
| nDs_019211459.1_38086 Very Low confidence | 17 | 32 | 17 |  | uuaucgauauauaauaacgaac | ucguuauuauauaucgauaaug |
| nDs_019211456.1_24325 Very Low confidence | 15 | 30 | 24 |  | aaaccagaucgacuacucaaca | uugaguaguugugcugguauu |
| nDs_019211460.1_41925 | 15 | 29 | 26 |  | uucgcccaucaccucugcaacu | cugcagaaugaaggccgaacgagaa |
| nDs_019211455.1_9409 Very Low confidence | 15 | 28 | 21 |  | uaacuugagcuuaaguguucugu | uggcacuuaaggucaagucagc |
| nDs_019211457.1_27698 | 14 | 39 | 36 |  | caucgcaacugucgacuggcu | ccaaacuucacacuugcagccca |
| nDs_019211455.1_14581 | 14 | 26 | 25 |  | uuaccuggguuuacguaaacuu | uuuacgaaaagccagauaag |
| nDs_019211460.1_42779 | 13 | 25 | 23 |  | ucuucauguacuuuccuuuuca | aaaggaaacuacacgaagaggcaca |
| nDs_019211460.1_45330 | 13 | 24 | 16 |  | gcuccgggcucgaucuguccu | aggacagaucgagcccggagcgcu |
| nDs_019211458.1_34592 | 13 | 23 | 19 |  | accguuguugucguugucgcc | gcggcaacaacaacaacggugacuc |
| nDs_019211454.1_96 | 12 | 22 | 19 |  | ucgucguugucguugucaccg | cggcagcgguaacggcgacagg |
| nDs_019211460.1_41659 | 12 | 23 | 21 |  | acgagauuguucuacuguguua | aacacaguagaacaaucucguugcu |
| nDs_019211454.1_4630 | 11 | 23 | 22 |  | cgccuauacuauaguagagc | cucuacuauaguauaggcu |
| nDs_019211455.1_9910 | 11 | 20 | 17 |  | aguccuaaucggaacuggaacca | gguuccaguuccgauuaggacua |
| nDs_019211457.1_28226 | 11 | 20 | 14 |  | agcuuuccacuaucgcuggagc | cgccagcgacgguggaaauacugc |
| nDs_019211460.1_43045 | 10 | 19 | 15 |  | gcuccgggcucgaucuguccu | ggacagaucgagcccgga |
| nDs_019211455.1_14279 | 10 | 27 | 18 |  | aguuauacgacagcuaucggc | ccgguagacuucguauacguuu |
| nDs_019211454.1_4561 | 9.8 | 16 | 8 |  | uguggacugugacgagcuagcc | cuagcucgucacaguccacacu |
| nDs_019211454.1_193 | 9.7 | 18 | 13 |  | ucgcaaugacaaauaagcucu | agcuuauucgucauugcga |
| nDs_019211454.1_3648 | 9.4 | 20 | 17 |  | aaagcacaucguuuaagcgu | cuaagacguuugcacacauu |
| nDs_019211456.1_19862 | 9 | 17 | 14 |  | aguucggcgagagugucuuugu | caaaguucucuccucguaccuu |
| nDs_019211456.1_23803 | 8.8 | 24 | 15 |  | agacggaucgaagaagcgcucc | accgcuucaccgauccguuugc |
| nDs_019211460.1_43961 | 8.5 | 16 | 15 |  | uauauacguccagauucgaaccc | uucgaauccggacguaaaca |
| nDs_019211457.1_27777 | 8.1 | 15 | 13 |  | guugcagcugaugugagugggu | ccccccacagcagcugcauuug |
| nDs_019211454.1_472 | 7.7 | 12 | 8 |  | uguggacugugacgagcuagcc | cuagcucgucacaguccacacu |
| nDs_019211455.1_10313 | 7.2 | 11 | 10 |  | agccgcacuuccuauggcagccu | cugccauaggaagugcggcu |
| nDs_019211455.1_15315 | 6.5 | 19 | 12 |  | agcuugcgaccgauacugcagc | ugcgguauuguucguaacgaggc |
| nDs_019211457.1_26766 | 6.4 | 10 | 9 |  | cgaugaccaagaauacgggagccu | gcucccguauucuuggucaucg |
| nDs_019211454.1_5704 | 6.4 | 12 | 11 |  | ucgcaaaaugaauuuuaccgu | gguaaaauucacuuugcgaua |
| nDs_019211455.1_9529 | 6.3 | 13 | 6 |  | acuguuuacguggagacggaacu | cuccgucucuacugucuaacagu |
| nDs_019211457.1_29060 | 5.9 | 11 | 9 |  | auccggacgaaguuuagaau | ucuaagcuucguccagcc |
| vde-miR-1010-5p | 5.8 | 92 | 92 | dme-miR-1010-5p | auaaguggcgauaucucgggau | cccgagauaucgccgcuuauau |
| nDs_019211456.1_23338 | 5.7 | 20 | 19 |  | agaacggagagaaacgaugcuc | agccucguacccuugcguucu |
| vde-miR-11-5p | 5.5 | 38 | 37 | dme-miR-11-5p | uaagaacucucugcacaauaaca | ugugcagagaguucucacgcc |
| vde-miR-4959-5p | 5.4 | 193 | 191 | dme-miR-4959-5p | auggccauaacgguuuagcagc | gcugcuaccgcuacggccauu |
| vde-miR-929-5p | 5.4 | 153 | 149 | dme-miR-929-5p | uaauugacuguugaaacaaaacu | uguuuuaacagucaacuacaau |
| vde-miR-307a-3p Low Confidence | 5.2 | 78945 | 78036 | dme-miR-307a-3p | ucacaaccuccuugagugagu | acucgcucaggagguagucgaug |
| vde-miR-318-3p Low Confidence | 5 | 15355 | 14384 | dme-miR-318-3p | ccacugggugagauucguccga | gcgaaucucucuacaguugcuu |
| vde-miR-307a-3p Low Confidence | 4.9 | 99225 | 86185 | dme-miR-307a-3p | ucacaaccuccuugagugagu | acuugcucaaucaguuuguggau |
| vde-miR-184-3p Low Confidence | 4.6 | 52965 | 52864 | dme-miR-184-3p | uggacggagaacugauaggg | cccuaucauucucuugucccgu |
| vde-miR-307a-3p Low Confidence | 4.6 | 99225 | 86185 | dme-miR-307a-3p | ucacaaccuccuugagugagu | acuugcucaaucaguuuguggau |
| vde-miR-4961-3p | 4.4 | 16 | 7 | dme-miR-4961-3p | uauguauauauauauauauauaua | uauauauauauauauaua |
| nDs_019211458.1_32852 | 4.3 | 8 | 6 |  | gaaacaacagcagcaauagcc | cuaaugcugucuguguuccgc |
| vde-miR-4984-5p | 3.9 | 481 | 472 | dme-miR-4984-5p | cgcgaauaugacaucgguacuga | ccaguaccugauguaauacucac |
| nDs_019211456.1_17117 | 2.5 | 94 | 94 |  | uaugcgaaucccaucgguugcu | caaccgauuggguucgcaugaa |
| nDs_019211459.1_40643 | 2.5 | 14 | 9 |  | uugcggaacagcgagcaaaaacu | uuugcucgcuguuccgcaaccu |
| nDs_019211459.1_38438 Very low confidence | 2.5 | 14 | 9 |  | uugcggaacagcgagcaaaaacu | uuugcucgcuguuccgcaaccu |
| nDs_019211457.1_28107 | 2.5 | 232 | 232 |  | auaagcggcgauaucucgggau | cccgagauaucgccacuuaugu |
| nDs_019211460.1_43069 Very Low Confidence | 2.4 | 863 | 862 |  | auggcgcugcauguaucgguuca | gauacaugcagcgccaucu |
| nDs_019211455.1_13999 | 2.3 | 12 | 12 |  | uagagguagaauuguuaacu | uuaacaauucuaccucuaac |
| nDs_019211455.1_9792 | 2.3 | 12 | 12 |  | uagagguagaauuguuaacu | uuaacaauucuaccucuaac |
| nDs_019211457.1_25599 | 2.3 | 95 | 95 |  | gccaccgguggcauagaugcu | caucuaugccaccggugguac |
| nDs_019211455.1_10445 | 2.3 | 19 | 19 |  | ucuuaacauaacgagugccauaga | uauggcacucguuauguuaagaua |
| nDs_019211460.1_43056 | 2.2 | 72 | 71 |  | aauguuacuuuaucgauccaac | uggaucgauaaaguaacauuca |
| nDs_019211459.1_38670 | 2.2 | 21 | 18 |  | aucgaccgaccgcuuuguccgc | gcggacaaagcggucggucga |
| nDs_019211460.1_45345 | 2.2 | 71 | 71 |  | aauguuacuuuaucgauccaac | uggaucgauaaaguaacauuca |
| nDs_019211457.1_28786 | 2.2 | 93 | 92 |  | accaccgguggcauagaugcuggcg | caucuaugccaccgguggca |
| nDs_019211456.1_18150 | 2.2 | 18 | 16 |  | acaucgagucgaagcggcggca | ugccgccgcuucgacucgauguu |
| nDs_019211454.1_1567 | 2.2 | 29 | 23 |  | cgcguuuaucgaagucgagacg | cgucucgacuucgauaaacgcg |
| nDs_019211456.1_22573 | 2.2 | 18 | 16 |  | acaucgagucgaagcggcggca | ugccgccgcuucgacucgauguu |
| nDs_019211457.1_28347 | 2.1 | 24 | 23 |  | cucggugugcaugguuugacuca | agucaaaccaugcacaccgagcu |
| nDs_019211459.1_40847 | 2.1 | 23 | 18 |  | aucgaccgaccgcuuuguccgc | gcggacaaagcggucggucga |
| nDs_019211454.1_1634 | 2.1 | 96 | 71 |  | cgcgagccuaucgauaguuagc | cuaucgauaggcucgcguuucgg |
| nDs_019211456.1_21124 | 2.1 | 12 | 12 |  | uauggcaguaaaacuuacucc | aguaaguuuuacugccauaau |
| nDs_019211454.1_1998 | 2.1 | 20 | 20 |  | gaugaugcuuucuucacauaca | uaugugaagaaagcaucauuuu |
| nDs_019211458.1_32365 | 2.1 | 83 | 72 |  | gcugcugcugcugcugcu | aguaacagcggcggcagc |
| nDs_019211456.1_19292 | 2.1 | 12 | 12 |  | ucuaugugaugcagaaucgccu | gcgauucuguaucacauagaua |
| nDs_019211456.1_23711 | 2.1 | 225 | 225 |  | ucuaugugauacagaaucgccu | gcgauucugcaucacauagaug |
| nDs_019211454.1_3175 | 2 | 148 | 148 |  | ugugccguugucggguaugcu | uauauucgacaaugucgcggg |
| nDs_019211458.1_33561 | 2 | 208 | 144 |  | uaaaccggucgugcgacauaca | gucgcacgaccgguuuaggcu |
| nDs_019211455.1_9834 | 2 | 210 | 210 |  | acagauuuauccaugagggcuc | gcccucauggauaaaucuguau |
| nDs_019211456.1_24398 | 2 | 12 | 12 |  | cguagcuuuccgcuguuguucug | ggggagcagcggaaagcuacacc |
| nDs_019211455.1_14041 | 2 | 202 | 202 |  | acagauuuauccaugagggcuc | gcccucauggauaaaucuguau |
| nDs_019211454.1_7060 Very Low Confidence | 2 | 87 | 77 |  | auagaacgacucucugguaggu | agagagucguucuaucauaacu |
| nDs_019211457.1_27725 | 2 | 982 | 609 |  | uccuucguaguuuggcaaagcac | uugccaaacuacgaagaacgcc |
| nDs_019211454.1_6280 | 2 | 19 | 13 |  | gcaagccugacugucugccagu | aggcaagcaggcaggcaggca |
| nDs_019211454.1_5638 | 1.9 | 28 | 24 |  | cgcguuuaucgaagucgagacg | cgucucgacuucgauaaacgcg |
| nDs_019211459.1_38428 | 1.9 | 16 | 16 |  | uggcgcgacaguuuaagaacagcu | cuguugaauugucgcgccaca |
| nDs_019211458.1_34548 | 1.9 | 20 | 18 |  | aggcuggauggcucgcugccu | gcacauaguccuagacugc |
| nDs_019211456.1_18675 | 1.9 | 35 | 27 |  | ugacgguagcggcagcaaaggg | auugcugcggccgccgucgccg |
| nDs_019211458.1_36880 | 1.9 | 18 | 18 |  | acguagcgcucucaagcugauuc | aucagcuugagagcgcuacgugu |
| nDs_019211460.1_41992 | 1.9 | 12 | 12 |  | uaauaaacgguuuuguugacu | ucgacaaaaccguuuauuagc |
| nDs_019211460.1_44383 | 1.9 | 13 | 13 |  | acggacaaacuauuuguagccu | gcuacaaguaguuuguccguuc |
| nDs_019211455.1_15480 | 1.9 | 45 | 45 |  | cagacagcucucugcacucagc | ucauugcagagagcugucugcu |
| nDs_019211456.1_21776 | 1.9 | 13 | 13 |  | cgggguauguauuuaguaaagcu | cuuuauuagaugcauaccccggg |
| nDs_019211457.1_30213 | 1.9 | 67 | 65 |  | cacugaggaaugacgguauacg | aguauaccgucauuccucagua |
| nDs_019211455.1_10783 | 1.9 | 14 | 14 |  | cacuaacugcuuuccugaacagu | uguuuaggaaagcacuuagugaa |
| nDs_019211458.1_36094 | 1.8 | 19 | 19 |  | gaaaaaaucgaacaagcugacu | ucagcuuguucgauuuuuucua |
| nDs_019211454.1_1662 | 1.8 | 13 | 13 |  | ucaauucacguaguacugagccu | guucaguacuacgugaauugaau |
| nDs_019211455.1_13946 | 1.8 | 14 | 14 |  | cgccauugaacuacucugugg | acagaguaguccaauggcgac |
| nDs_019211455.1_15579 | 1.8 | 105 | 103 |  | uucccgaucagggcccccugga | caaugggcccaucgccggggccac |
| nDs_019211456.1_21938 | 1.8 | 1701 | 1590 |  | auuaugaucgauuugaauggccc | gcuucgucaucauaauau |
| nDs_019211459.1_40272 | 1.8 | 38 | 6 |  | cauaugacucuucgcuucgucuu | aaagcgaagagucauauguaga |
| nDs_019211455.1_8719 Very Low confidence | 1.8 | 43 | 34 |  | uggccguagcgguagcagcug | ugcugcuaaaccguuauggc |
| nDs_019211456.1_17457 | 1.8 | 1701 | 1590 |  | auuaugaucgauuugaauggccc | gcuucgucaucauaauau |
| nDs_019211457.1_25248 | 1.7 | 9 | 8 |  | ugccagcaucgggccguucgaag | cgacgggucgcgaaugcuggcaau |
| nDs_019211457.1_26906 | 1.7 | 22 | 22 |  | uagauauaguccucugucgaag | ucgacaaagcacuauaucuaua |
| nDs_019211454.1_6096 | 1.7 | 144 | 141 |  | ucgucgucggugguaacggc | gcugcuaccgcuuaugaugcc |
| nDs_019211456.1_17791 | 1.7 | 18 | 18 |  | uauauaagaauacaacuuggcg | ccaaguuguauucuuauauaga |
| nDs_019211456.1_23888 | 1.7 | 17 | 12 |  | uagcuagaagcuagauugcu | agcaaucuagcuucuagcuaua |
| nDs_019211454.1_6002 | 1.7 | 23 | 23 |  | uucugauacgacgcggacccuu | cugucugcgucguuucagccaug |
| nDs_019211459.1_37201 | 1.7 | 25 | 23 |  | auaaacaauagggaagacucaaag | ucccuauuguuuauaacc |
| nDs_019211455.1_15581 | 1.7 | 56 | 56 |  | aauaacaaguaacuuauagacg | ucuauaaguugcuuguuauuau |
| nDs_019211454.1_2659 | 1.7 | 20 | 20 |  | aauuggucuguguggagg | ucuugccggccaagcag |
| nDs_019211460.1_45599 | 1.7 | 52 | 52 |  | acauucgcgaucacucgugacc | ucacgagugaucgcgaaaauuc |
| nDs_019211454.1_1816 Very Low Confidence | 1.7 | 77 | 72 |  | cacgguuacguccucuugugcu | caaggggacguaaccguauagua |
| nDs_019211457.1_27368 | 1.7 | 14 | 14 |  | agcggaccaaugaaguacu | uacuucauugguccgcuag |
| nDs_019211456.1_18867 | 1.7 | 85 | 85 |  | uugucgcaacucuugccgagcug | uaccggcaacaguugcaacagca |
| nDs_019211460.1_43946 | 1.6 | 44 | 36 |  | ugccaaaacguugggaugauguu | gguuccaacguuuuggcagguu |
| nDs_019211457.1_29829 Low confidence | 1.6 | 29033 | 28510 |  | ucggcucuagcaaucaucacaau | aauguguugauagcagagccg |
| nDs_019211456.1_19534 | 1.6 | 12 | 11 |  | aagagcuguaaagaugaagcu | uucucgauucgcacgcuuucc |
| nDs_019211458.1_33253 | 1.5 | 21 | 21 |  | gaaaaaaucgaacaagcugacu | ucagcuuguucgauuuuuucua |
| nDs_019211455.1_9685 | 1.5 | 20 | 16 |  | uuucgagcuggcaucuaacuugu | ucagaugccagcucgaaacu |
| nDs_019211457.1_25551 | 1.5 | 24 | 24 |  | aauauaauuucguuuucgaagg | uucgaaaacgaaauuauauuuu |
| nDs_019211455.1_9027 Very Low confidence | 1.5 | 109 | 84 |  | aaucuagcggcguuucggcuga | caaagcgccgcucuagaaacgc |
| nDs_019211455.1_11854 | 1.5 | 13 | 13 |  | cacggcacuucucaacuucacu | uguuguugcaagugccguugc |
| nDs_019211456.1_17176 | 1.5 | 31 | 21 |  | agaaacuugugcuguucuuggaau | caaagauggcgcauguuucca |
| nDs_019211457.1_29650 | 1.5 | 11 | 11 |  | uaggccaugugccaaaaggaugau | cgccuuuuggcaaauggccugga |
| nDs_019211458.1_33664 | 1.5 | 80 | 80 |  | auagagauuuauguacuugcu | caaguacauaaaucucuauuu |
| nDs_019211460.1_43980 | 1.5 | 13 | 13 |  | aguacguuggacggugccaa | ggcgccgcgacuccagcugcugc |
| nDs_019211459.1_38309 | 1.5 | 128 | 125 |  | uacgcugacacgguacagacu | cgucugucguguuaggcgcagc |
| nDs_019211455.1_12422 | 1.5 | 252 | 252 |  | acggacugcggcaucuuggugu | acaaagaugggcaguucaauuuuguca |
| nDs_019211460.1_44454 | 1.5 | 70 | 61 |  | gcugcugcugcugcugcu | caguaugugcaguagcucua |
| nDs_019211459.1_37290 | 1.4 | 35 | 35 |  | agcgaccgucgucacggacagc | ugcuguuucugcagccgcugg |
| nDs_019211454.1_1794 | 1.4 | 72 | 72 |  | uagauauuucuugaucagacu | ccugaccaacaaauaucuauc |
| nDs_019211454.1_1917 | 1.4 | 113 | 112 |  | uuaggugagagucaguguaguu | aacuacacugacuuucauauga |
| nDs_019211456.1_18369 | 1.4 | 21 | 19 |  | uuuauuuuugccauagccaca | auaggcuauggcaaaaauaaac |
| nDs_019211454.1_4763 | 1.4 | 32 | 32 |  | uauauaauucgcgaaugugccu | gcacauucgcgaauuauauauu |
| nDs_019211454.1_2475 | 1.4 | 22 | 22 |  | guugucgauggcuauccag | ggaagaggccaucagcacug |
| nDs_019211458.1_32553 | 1.4 | 181 | 181 |  | uugcccguucguucgcugaau | aaagcgagcaggcgggcggcc |
| nDs_019211455.1_14113 | 1.3 | 31 | 23 |  | aguagacgcaguaagguguaga | cacuucaccgcguccacuuauu |
| nDs_019211455.1_15769 | 1.3 | 47 | 47 |  | uccaaaggagucgaggucgucuu | gacgcccuccucccaagaauu |
| nDs_019211454.1_2415 | 1.3 | 60 | 60 |  | gaaugcaucuaggagacugaau | ucagucuccuagaugcauacau |
| nDs_019211454.1_1626 | 1.3 | 16 | 16 |  | uuuagaugcuucuccacguagcu | ccacguggagaagcagcuuagaac |
| nDs_019211454.1_6476 | 1.3 | 653 | 652 |  | uaugcaucuaggagacugaca | auucagucuccuagaugcauuc |
| nDs_019211454.1_5883 | 1.2 | 20 | 20 |  | ucagcaaaacgcuucguggaac | ucugugaaaguuuugccgcuc |
| nDs_019211458.1_35574 | 1.2 | 144 | 144 |  | augcggcuguagccacccuaccu | gugggauguuggcuguugccacaucu |
| nDs_019211459.1_37910 | 1.2 | 12 | 10 |  | aacggcgaucauuaggucc | gcuugaacuaucugcugcgug |
| nDs_019211460.1_41557 | 1.2 | 15 | 15 |  | auagguccgaggcugugcuccu | gaguacaccuucggucuauag |
| nDs_019211460.1_42621 | 1.2 | 86 | 86 |  | cacgaugaugcguugagggu | cuguagcacauuuaacauauccgcu |
| nDs_019211457.1_31348 | 1.2 | 72 | 72 |  | ugccgggcugcuagaagaagaaga | uacuucuucuaaccccgcgcuaa |
| nDs_019211454.1_4014 | 1.1 | 24 | 24 |  | aagucuugucuaguaguaagcu | cuuacuauuaggcaagagguaa |
| nDs_019211457.1_28742 | 1.1 | 30 | 30 |  | aauauaauuucguuuucgaagg | uucgaaaacgaaauuauauucg |
| nDs_019211455.1_15756 | 1.1 | 25 | 24 |  | ucgaugaagcugauucgauacacu | gguggaaucagcuguccucguag |
| nDs_019211457.1_30566 | 1.1 | 10 | 7 |  | ucugacacgcugcaauugcg | ucugcaagugugaaagccu |
| nDs_019211458.1_36783 | 1.1 | 19 | 19 |  | aguagugaacaauucaaugggc | ccauugaaucggucacaaaucg |
| nDs_019211459.1_40795 | 1 | 18 | 17 |  | ugucgucugcucucauuugcc | ggugggugggcagacguccggu |
| nDs_019211455.1_15030 | 1 | 10 | 10 |  | ugaaaagaaaaaaauuaggacu | uccuaauuuuuuucuuuucaua |
| nDs_019211455.1_11830 | 1 | 12 | 11 |  | uaguggaaccucgucugcggaga | uucgcagggaggucuccaacc |
| nDs_019211460.1_43035 | 1 | 10 | 10 |  | ucagaaggcagcuuagucggua | ccgacuaagcugccuucugaua |
| nDs _019211456.1_19171 | 1 | 26 | 26 |  | auuuuuacuaaguucuuguccu | gacaaaaacuuaguaaaagcgc |

**Table S3.** Sex-specific predicted miRNAs in *Varroa* mites (other details are provided in Table S1).

| **Male specific miRNAs** | **Female specific miRNAs** |
| --- | --- |
| nDS_019211455.1_10989 | nDS_019211455.1_10999 |
| nDS_019211455.1_16072 | nDS_019211459.1_37116 |
|  | nDS_019211455.1_10993 |

**Table S4.** Orthologs of *Apis mellifera* (ame), *Ixodes scapularis* (isc), and other arthropod miRNAs detected in miRDeep2 analysis.

| **tag id** | **Score** | **ame miRNA** | **miRNA with the same seed** | **consensus mature seq** |
| --- | --- | --- | --- | --- |
| **NC_037651.1_25019** | **2.40E+06** | **ame-bantam-3p** | **isc-bantam-MIMAT0012679** | **ugagaucauugugaaagcugauu** |
| **NC_037648.1_21730** | **3.70E+05** | **ame-miR-8-3p** | **isc-miR-8-MIMAT0012706** | **uaauacugucagguaaagauguc** |
| **NC_037638.1_3193** | **9.60E+04** | **ame-miR-9a-5p** |  | **ucuuugguuaucuagcuguauga** |
| **NC_037653.1_27376** | **9.10E+04** | **ame-miR-10-5p** | **isc-miR-10-MIMAT0012682** | **uacccuguagauccgaauuugu** |
| **NC_037641.1_8156** | **7.80E+04** | **ame-miR-305-5p** | **isc-miR-305-MIMAT0018920** | **auuguacuucaucaggugcucu** |
| **NC_037639.1_4833** | **3.70E+04** | **ame-miR-12-5p** | **isc-miR-12-MIMAT0012684** | **ugaguauuacaucagguacuggu** |
| **NC_037640.1_6986** | **3.40E+04** | **ame-miR-375-3p** | **isc-miR-375-MIMAT0012700** | **uuuguucguucggcucgaguua** |
| **NC_037638.1_1980** | **2.20E+04** | **ame-miR-2-3p** | **isc-miR-2a-MIMAT0012696** | **uaucacagccagcuuugaugagc** |
| **NC_037642.1_9724** | **2.10E+04** | **ame-miR-34-5p** |  | **uggcaguguuguuagcugguugu** |
| **NC_037647.1_18492** | **2.10E+04** | **ame-miR-263a-5p** | **isc-miR-263a-MIMAT0012692** | **aauggcacuggaagaauucacgg** |
| **NC_037638.1_1972** | **1.90E+04** | **ame-miR-2-3p** | **isc-miR-2a-MIMAT0012696** | **uaucacagccagcuuugaugagc** |
| **NC_037641.1_8153** | **1.90E+04** | **ame-miR-275-3p** | **isc-miR-275-MIMAT0012693** | **ucagguaccugaaguagcgcgc** |
| **NC_037638.1_1981** | **1.80E+04** | **ame-miR-71-5p** | **isc-miR-71-MIMAT0012703** | **ugaaagacauggguagugagaug** |
| **NC_037644.1_14692** | **1.40E+04** | **ame-miR-276-3p** | **isc-miR-276-MIMAT0018919** | **uaggaacuucauaccgugcucu** |
| **NC_037650.1_24048** | **6.60E+03** | **ame-miR-6001-5p** |  | **uucucuuugguuguuaccacuag** |
| **NC_037639.1_4040** | **5.60E+03** | **ame-miR-315-5p** | **isc-miR-315-MIMAT0012698** | **uuuugauuguugcucagaaagc** |
| **NC_037653.1_27491** | **3.40E+03** | **ame-miR-1-3p** | **isc-miR-1-MIMAT0012681** | **uggaauguaaagaaguauggag** |
| **NC_037645.1_16261** | **1.80E+03** | **ame-let-7-5p** | **isc-let-7-MIMAT0012680** | **ugagguaguagguuguauagu** |
| **NC_037652.1_26128** | **1.60E+03** | **ame-miR-306-5p** | **isc-miR-275-MIMAT0012693** | **ucagguacugagugacucugagu** |
| **NC_037638.1_1976** | **5.50E+02** | **ame-miR-13b-3p** | **isc-miR-2a-MIMAT0012696** | **uaucacagccauuuuugacgauu** |
| **NC_037645.1_16263** | **3.00E+02** | **ame-miR-100-5p** |  | **acccguagauccgaacuugugg** |
| **NC_037647.1_18639** | **2.30E+02** | **ame-miR-3785-3p** | **isc-miR-10-MIMAT0012682** | **uacccuguaacguccugagacu** |
| **NC_037646.1_17866** | **2.00E+02** | **ame-miR-11-3p** | **isc-miR-2a-MIMAT0012696** | **caucacaggcagaguucuaguu** |
| **NC_037638.1_1978** | **1.70E+02** | **ame-miR-13a-3p** | **isc-miR-2a-MIMAT0012696** | **uaucacagccauuuugaugagcu** |
| **NC_037639.1_5002** | **1.70E+02** | **ame-miR-279c-3p** | **isc-miR-279-MIMAT0012694** | **ugacuagagucacacucgucc** |
| **NC_037652.1_26159** | **1.60E+02** | **ame-miR-92c-5p** |  | **aauugcacucgucccggccugc** |
| **NC_037653.1_27858** | **1.40E+02** | **ame-miR-993-3p** | **isc-miR-993-MIMAT0012709** | **gaagcucgucucuacagguaucu** |
| **NC_037652.1_26130** | **1.30E+02** | **ame-miR-79-3p** |  | **cuuugguaauauagcucuauga** |
| **NW_020555859.1_28078** | **8.10E+01** | **ame-miR-750-3p** | **isc-miR-750-MIMAT0012704** | **ccagaucuaacucuuccagcuca** |
| **NC_037639.1_4835** | **7.60E+01** | **ame-miR-3477-5p** | **isc-miR-5307-MIMAT0021377** | **uaaucucaugcgguaacugugagu** |
| **NC_037638.1_3217** | **7.60E+01** | **ame-miR-281-3p** | **dme-miR-281-2-5p** | **aagagagcuauccaucgacagu** |
| **NC_037645.1_15726** | **6.80E+01** | **ame-miR-278-3p** | **dme-miR-278-5p** | **ccggaugaggucuucaucgacc** |
| **NC_037639.1_5423** | **4.30E+01** | **ame-miR-3715-5p** | **ame-miR-3715-5p-MIMAT0018597** | **ucgguaagcagaguauaagaccu** |
| **NC_037645.1_15480** | **4.10E+01** | **ame-miR-927a-5p** | **dme-miR-927-5p** | **uuuagaauuccuacgcuuuacc** |
| **NC_037641.1_7964** | **3.50E+01** | **ame-miR-263b-5p** | **isc-miR-96-MIMAT0012708** | **cuuggcacuggaagaauucacagau** |
| **NC_037640.1_7080** | **2.20E+01** | **ame-miR-3718a-3p** | **ame-miR-3718a-3p-MIMAT0018520** | **uccccuguccugucccgauag** |
| **NC_037651.1_25269** | **1.70E+01** | **ame-miR-3786-5p** | **ame-miR-3786-5p-MIMAT0018565** | **cuuguccugguucauguagggc** |
| **NC_037640.1_7124** | **1.60E+01** | **ame-miR-3719-3p** | **ame-miR-3719-3p-MIMAT0018531** | **uacggauugcgugacuuuucg** |
| **NC_037638.1_1799** | **1.30E+01** | **ame-miR-33-5p** |  | **caauacuucuacagugcaacu** |
| **NC_037651.1_24868** | **1.20E+01** | **ame-miR-282-5p** | **dme-miR-282-5p** | **uagccucuccuaggcuuugucu** |
| **NW_020555859.1_28076** | **8.9** | **ame-miR-1175-3p** | **ame-miR-1175-3p-MIMAT0018507** | **ugagauucacuccuccaacuuac** |
| **NC_037644.1_13325** | **7.3** | **ame-miR-3049-5p** | **ame-miR-3049-3p-MIMAT0018528** | **uccguccaacuccuuuccgucu** |
| **NC_037642.1_9717** | **5.5** | **ame-miR-317-3p** | **isc-miR-317-MIMAT0012699** | **ugaacacagcuggugguaucu** |
| **NC_037640.1_7082** | **5.5** | **ame-miR-3718c-5p** | **ame-miR-3718a-3p-MIMAT0018520** | **uccccuguccugucccgauag** |
| **NC_037638.1_1974** | **5.5** | **ame-miR-2-3p** | **isc-miR-2a-MIMAT0012696** | **uaucacagccagcuuugaugagc** |
| **NC_037650.1_24308** | **5.4** | **ame-miR-87-3p** | **isc-miR-87-MIMAT0012707** | **gugagcaaaguuucaggugugu** |
| **NC_037638.1_2038** | **5.3** | **ame-miR-193-3p** | **ame-miR-193-3p-MIMAT0018508** | **uacuggccugcuaagucccaag** |
| **NC_037641.1_8031** | **5.3** | **ame-miR-2796-3p** | **ame-miR-2796-3p-MIMAT0018591** | **guaggccggcggaaacuacuugc** |
| **NC_037648.1_21038** | **5.2** | **ame-miR-14-3p** | **ame-miR-14-3p-MIMAT0004423** | **ucagucuuuuucucucuccuau** |
| **NC_037644.1_14179** | **5.2** | **ame-miR-1000-5p** | **ame-miR-1000-5p-MIMAT0010119** | **auauugucuugucacagcagu** |
| **NC_037645.1_16259** | **5.1** | **ame-miR-125-5p** | **ame-miR-125-5p-MIMAT0001474** | **ccccugagacccuaacuuguga** |
| **NC_037638.1_18** | **5.1** | **ame-miR-2b-5p** | **dme-miR-2a-2-5p** | **gcucaucaaagcuggcugugaua** |
| **NC_037642.1_9721** | **5** | **ame-miR-277-3p** | **ame-miR-277-3p-MIMAT0001481** | **uaaaugcacuaucugguacgac** |
| **NC_037639.1_4325** | **5** | **ame-miR-210-3p** | **dme-miR-210-3p** | **cuugugcgugugacagcggcu** |
| **NC_037639.1_4837** | **5** | **ame-miR-283-5p** | **ame-miR-6041-3p-MIMAT0023663** | **aaauaucagcugguaauucuggga** |
| **NC_037646.1_17257** | **5** | **ame-miR-92b-3p** | **ame-miR-92b-3p-MIMAT0010117** | **aauugcacccgucccggccu** |
| **NC_037642.1_9989** | **5** | **ame-miR-996-3p** | **isc-miR-279-MIMAT0012694** | **ugacuagauacauacucgucu** |
| **NC_037646.1_17278** | **5** | **ame-miR-7-5p** | **isc-miR-7-MIMAT0012702** | **uggaagacuagugauuuuguuguu** |
| **NC_037645.1_16137** | **5** | **ame-miR-31a-5p** | **dme-miR-31b-5p** | **aggcaagaugucggcauagcuga** |
| **NC_037646.1_17827** | **5** | **ame-miR-2765-5p** | **ame-miR-2765-5p-MIMAT0023551** | **ugguaacuccaccaccguuggc** |
| **NC_037648.1_20833** | **4.9** | **ame-miR-219-5p** | **isc-miR-219-MIMAT0012690** | **ugauuguccaaacgcaauucuug** |
| **NC_037652.1_26157** | **4.9** | **ame-miR-92a-3p** | **ame-miR-92b-3p-MIMAT0010117** | **uauugcacuugucccggccuau** |
| **NC_037651.1_24741** | **4.9** | **ame-miR-137-3p** | **ame-miR-137-3p-MIMAT0004421** | **uuauugcuugagaauacacgu** |
| **NC_037645.1_15377** | **4.8** | **ame-miR-29b-3p** | **isc-miR-285-MIMAT0012695** | **uagcaccauuugaaaucaguac** |
| **NC_037652.1_26458** | **4.8** | **ame-miR-279b-3p** | **isc-miR-279-MIMAT0012694** | **ugacuagaucgaaauacucguc** |
| **NC_037650.1_23738** | **4.8** | **ame-miR-190-5p** | **ame-miR-190-5p-MIMAT0004440** | **agauauguuugauauucuugguu** |
| **NC_037653.1_27505** | **4.7** | **ame-miR-133-3p** | **isc-miR-133-MIMAT0012686** | **uugguccccuucaaccagcugu** |
| **NC_037652.1_26797** | **4.7** | **ame-miR-252a-5p** | **ame-miR-252a-5p-MIMAT0010113** | **auaaguacuagugccgcagga** |
| **NC_037642.1_9718** | **4.7** | **ame-miR-317-3p** | **isc-miR-317-MIMAT0012699** | **ugaacacagcuggugguaucu** |
| **NC_037638.1_2036** | **4.6** | **ame-miR-2788-3p** | **isc-miR-5309-MIMAT0021379** | **caaugcccuucgaaauccca** |
| **NC_037652.1_26801** | **4.5** | **ame-miR-929-5p** |  | **aauugacucuaguagggagu** |
| **NC_037652.1_26460** | **4.5** | **ame-miR-3791-3p** | **ame-miR-3791-3p-MIMAT0018570** | **ucaccggguaggauucaucca** |
| **NC_037650.1_24184** | **3.5** | **ame-miR-3783-3p** | **ame-miR-3783-3p-MIMAT0018561** | **uacuuucaauuguuugaugagg** |
| **NC_037652.1_26799** | **2.5** | **ame-miR-252b-5p** | **isc-miR-252b-MIMAT0012691** | **uuaaguaguagugucguagauga** |
| **NC_037638.1_848** | **2.1** | **ame-miR-3770-5p** | **ame-miR-3770-5p-MIMAT0018545** | **aauccugcaucaagugcguuguu** |
| **NC_037638.1_986** | **2.1** | **ame-miR-6039-5p** | **ame-miR-6039-5p-MIMAT0023659** | **aaucgaacgcgugaguuuacgu** |
| **NC_037639.1_4324** | **1.8** | **ame-miR-6038-5p** | **ame-miR-6038-5p-MIMAT0023658** | **uauguuucugucuuauuucauu** |
| **NC_037638.1_2330** | **1.7** | **ame-miR-307-3p** |  | **cacaaccuuuuugagugagcga** |
| **NC_037646.1_17167** | **1.5** | **ame-miR-279d-3p** |  | **ugacuagauccacacucauc** |
| **NC_037652.1_26454** | **1.3** | **ame-miR-2944-3p** | **isc-miR-2a-MIMAT0012696** | **uaucacagcaguaguuaccuggu** |
| **NC_037648.1_20569** | **1.2** | **ame-miR-6000b-3p** | **ame-miR-6000b-3p-MIMAT0023661** | **uagagacgaguaguacccacgagu** |
| **NC_037642.1_9987** | **1.1** | **ame-miR-279a-3p** |  | **ugacuagauccacacucauu** |
| **NC_037640.1_6511** | **1** | **ame-miR-6047a-5p** | **ame-miR-6047a-5p-MIMAT0037456** | **aguggacugacuggccugugcu** |
| **NC_037638.1_3427** | **0.9** | **ame-miR-932-5p** | **ame-miR-932-5p-MIMAT0004444** | **ucaauuccguagugcauugcag** |
| **NC_037639.1_4123** | **0.6** | **ame-miR-981-3p** | **ame-miR-981-3p-MIMAT0010121** | **uucguugucaacgaaaccugca** |
| **NC_037652.1_26456** | **0.6** | **ame-miR-9c-3p** | **ame-miR-79-3p-MIMAT0004433** | **uaaagcuaguucagcaaggc** |
| **NC_037641.1_8682** | **0.6** | **ame-miR-6012-3p** | **ame-miR-6012-3p-MIMAT0023670** | **uucggcgaugagaucagccuguc** |
| **NC_037648.1_21662** | **0.2** | **ame-miR-6000a-5p** | **ame-miR-6000a-3p-MIMAT0023540** | **uaguacgagaaguacccacaa** |
| **NC_037640.1_7123** | **0.1** | **ame-miR-3719-3p** | **ame-miR-3719-3p-MIMAT0018531** | **uacggauugcgugacuuuucg** |
| **NC_037638.1_2037** | **-0.3** | **ame-miR-193-3p** | **ame-miR-193-3p-MIMAT0018508** | **uacuggccugcuaagucccaag** |
| **NC_037650.1_24307** | **-1** | **ame-miR-87-3p** | **isc-miR-87-MIMAT0012707** | **gugagcaaaguuucaggugugu** |
| **NC_037653.1_27506** | **-1.2** | **ame-miR-133-3p** | **isc-miR-133-MIMAT0012686** | **uugguccccuucaaccagcugu** |
| **NC_037640.1_5771** | **-1.8** | **ame-miR-6043-3p** | **ame-miR-6043-3p-MIMAT0023665** | **auggugaccgugaucuauucc** |
| **NC_037641.1_8154** | **-2.1** | **ame-miR-275-3p** | **isc-miR-275-MIMAT0012693** | **ucagguaccugaaguagcgcgc** |
| **NC_037648.1_20834** | **-2.3** | **ame-miR-219-5p** | **isc-miR-219-MIMAT0012690** | **ugauuguccaaacgcaauucuug** |
| **NC_037652.1_25828** | **-2.5** | **ame-miR-927b-5p** |  | **uuuagaauuuguacgcucugu** |
| **NC_037642.1_10552** | **-3.4** | **ame-miR-9888-5p** |  | **guucuaugcuugaccaucauu** |
| **NC_037648.1_21441** | **-3.7** | **ame-miR-6067-5p** |  | **acggaucaagcuuuuugug** |
| **NC_037651.1_25020** | **-6.3** | **ame-bantam-3p** | **isc-bantam-MIMAT0012679** | **ugagaucauugugaaagcugauu** |
| **NC_037652.1_26127** | **-1.30E+01** | **ame-miR-306-5p** | **isc-miR-275-MIMAT0012693** | **ucagguacugagugacucugagu** |

# Supplementary Figures


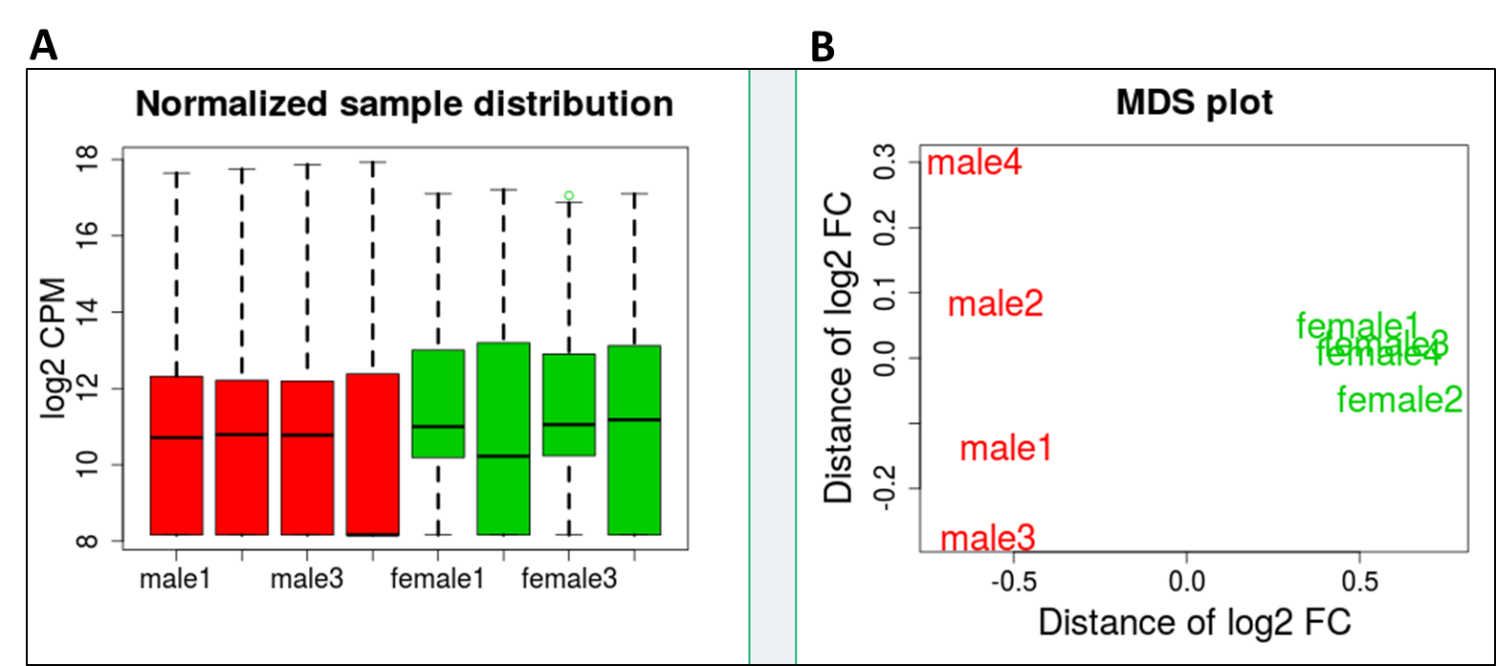


**Supplementary Figure S1.** A. Normalized sample distribution for male and female varroa samples. B. Multidimensional Scaling (MDS) plot shows variation in differential expression among male samples, distance between sample labels indicates dissimilarity in log2 Fold change (FC).
